# TransCode: Uncovering COVID-19 transmission patterns via deep learning

**DOI:** 10.1186/s40249-023-01052-9

**Published:** 2023-02-28

**Authors:** Jinfu Ren, Mutong Liu, Yang Liu, Jiming Liu

**Affiliations:** Department of Computer Science, Hong Kong Baptist University, Hong Kong SAR, China

**Keywords:** COVID-19, Densely populated regions, Spatiotemporal transmission dynamics and heterogeneity, Meta-population, Human mobility and contact behaviors, TransCode, Deep transfer learning

## Abstract

**Background:**

The heterogeneity of COVID-19 spread dynamics is determined by complex spatiotemporal transmission patterns at a fine scale, especially in densely populated regions. In this study, we aim to discover such fine-scale transmission patterns via deep learning.

**Methods:**

We introduce the notion of TransCode to characterize fine-scale spatiotemporal transmission patterns of COVID-19 caused by metapopulation mobility and contact behaviors. First, in Hong Kong, China, we construct the mobility trajectories of confirmed cases using their visiting records. Then we estimate the transmissibility of individual cases in different locations based on their temporal infectiousness distribution. Integrating the spatial and temporal information, we represent the TransCode via spatiotemporal transmission networks. Further, we propose a deep transfer learning model to adapt the TransCode of Hong Kong, China to achieve fine-scale transmission characterization and risk prediction in six densely populated metropolises: New York City, San Francisco, Toronto, London, Berlin, and Tokyo, where fine-scale data are limited. All the data used in this study are publicly available.

**Results:**

The TransCode of Hong Kong, China derived from the spatial transmission information and temporal infectiousness distribution of individual cases reveals the transmission patterns (e.g., the imported and exported transmission intensities) at the district and constituency levels during different COVID-19 outbreaks waves. By adapting the TransCode of Hong Kong, China to other data-limited densely populated metropolises, the proposed method outperforms other representative methods by more than 10% in terms of the prediction accuracy of the disease dynamics (i.e., the trend of case numbers), and the fine-scale spatiotemporal transmission patterns in these metropolises could also be well captured due to some shared intrinsically common patterns of human mobility and contact behaviors at the metapopulation level.

**Conclusions:**

The fine-scale transmission patterns due to the metapopulation level mobility (e.g., travel across different districts) and contact behaviors (e.g., gathering in social-economic centers) are one of the main contributors to the rapid spread of the virus. Characterization of the fine-scale transmission patterns using the TransCode will facilitate the development of tailor-made intervention strategies to effectively contain disease transmission in the targeted regions.

**Graphical Abstract:**

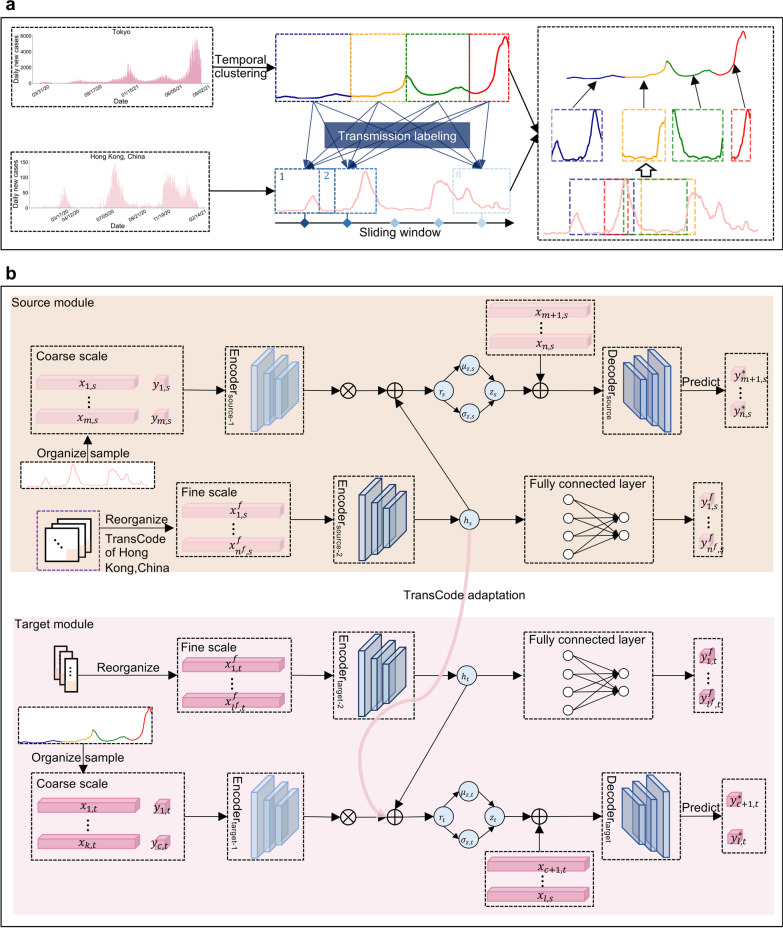

**Supplementary Information:**

The online version contains supplementary material available at 10.1186/s40249-023-01052-9.

## Background

**Fig. 1:**
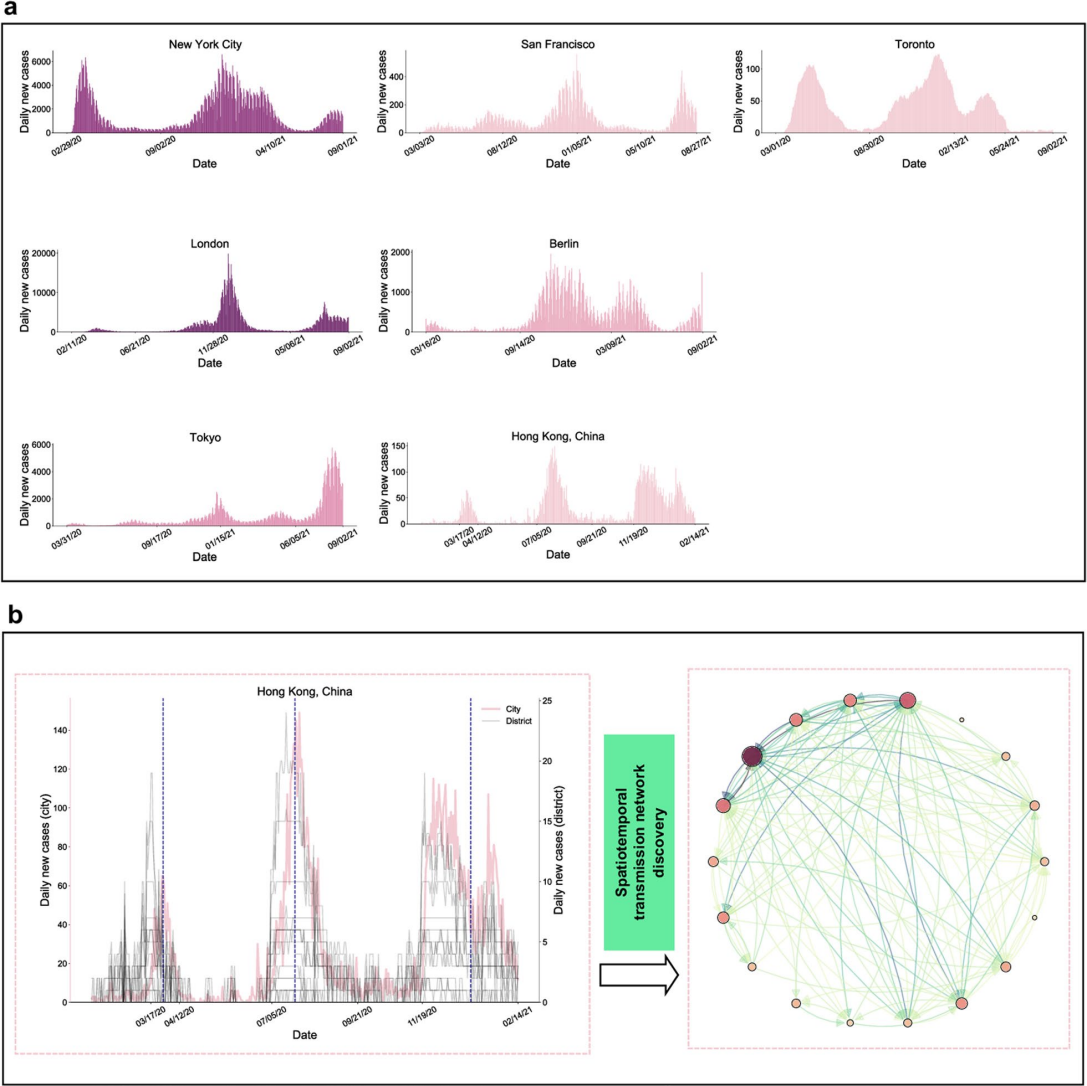
Global COVID-19 transmission dynamics. **a** The subfigures display the daily infection dynamics in seven representative metropolises: New York City, San Francisco, Toronto, London, Berlin, Tokyo, and Hong Kong, China. **b** The detailed COVID-19 transmission dynamics in Hong Kong, China. The left box shows the city-level daily infection curve (pink) and 18 district-level daily infection curves (grey). The right box displays the cross-district transmission network that we aim to infer in this study. Each node of the network denotes a district, with darker colors representing districts with higher transmission risks. The links between nodes denote the cross-district transmission intensity, with darker colors representing higher transmission intensities

Coronavirus disease 2019 (COVID-19) has rapidly spread worldwide since January 2020. As of December 5, 2022, more than 639 million cases and more than 6.6 million deaths have been confirmed by the World Health Organization (WHO) [1]. Understanding disease transmission dynamics is essential for developing and implementing appropriate intervention strategies to effectively control the COVID-19 pandemic [2–9]. However, COVID-19 transmission dynamics are difficult to capture and quantify due to geographic (e.g., metropolises and regions) and temporal (e.g., days, weeks, or months) heterogeneity. As shown in Fig. 1a, COVID-19 dynamics differ in the seven representative metropolises (New York City, San Francisco, Toronto, London, Berlin, Tokyo, and Hong Kong, China) investigated in this study. Moreover, the dynamics within each metropolis show clear temporal heterogeneity; the outbreak wave durations range from days to months, and the wave peaks (in terms of daily infection numbers) vary from dozens to hundreds and from thousands to tens of thousands.

The heterogeneity of disease dynamics is determined by complex spatiotemporal transmission patterns at a fine scale [9–14], especially in densely populated regions. Figure 1b (left) displays the COVID-19 transmission patterns in Hong Kong, China at a fine scale (the district level). The fine-scale transmission patterns dramatically vary in both spatial and temporal dimensions, as demonstrated by the diversity among the grey curves (which show the district-level daily infections) in the top left of Fig. 1b. Moreover, Fig. 1b (right) shows the complex interactions among districts, representing human mobility and contact behaviors at the metapopulation level, which are further analyzed and discussed in later sections. The spatiotemporal variability and complex interactions across different districts, although central to the understanding of disease transmission, are difficult to capture and quantify.

To understand disease transmission dynamics to facilitate effective disease control, we need to answer a critical question: How can we quantitatively characterize the underlying spatiotemporal transmission patterns of COVID-19 at the metapopulation level to accurately capture transmission dynamics and predict future risks, especially in densely populated regions? Specifically, we have to address two technical challenges:

1. How can we uncover and represent fine-scale cross-district transmission patterns using available data?
2. How can we adapt the transmission patterns to other regions where fine-scale data are limited?

Existing measurements or statistics of disease dynamics, such as the basic reproduction number ${R}_{0}$ or the daily infection number, can provide an overall evaluation of disease transmission severity in a specific region [15–18]. However, such coarse measurements conceal a considerable amount of spatial heterogeneity and temporal variability at fine resolutions (e.g., the district level in a city), which are crucial for depicting disease dynamics. Several recent works have recognized the importance of characterizing fine-scale transmission patterns [19–24]. However, these works mainly have focused on qualitative characterizations, such as spatial heterogeneity at the tract level [19–21] or transmission clusters within different scenarios [19, 22–24]. Without establishing a clear quantitative connection between fine-scale transmission patterns and city-level disease dynamics, it is still unclear how fine-scale information may be useful for quantifying and inferring disease dynamics.

In addition to investigating disease transmission from an epidemiological perspective, some researchers have modeled and inferred disease dynamics using computational methods. These methods can be categorized into two categories: mechanism-based methods and data-driven methods. Mechanism-based methods simulate disease dynamics by assuming that transmission follows some pre-defined models (e.g., mechanism or compartment models), which are determined using a set of ordinary differential equations (ODEs) [9, 25–29]. Mechanism-based methods have improved the quantitative characterization of disease transmission; however, ODE-governed assumptions might not hold in reality, thus limiting the practical applicability of such methods. Although some works [27–29] have incorporated human mobility data into ODEs to yield predictions, the analyses were conducted at the city level and lacked the consideration of fine-scale information. Data-driven methods (e.g., deep learning methods) [14, 30, 31] generally use historical data (e.g., infection numbers) to train models for future predictions. However, these methods do not incorporate fine-scale transmission information, resulting in inaccurate and unexplainable prediction results, especially when available data for model training is limited. Some other methods adopt transfer learning to improve the accuracy of prediction in data-limited regions [32–34]. Gautam et al. directly utilize the data obtained from data-rich countries (in which the epidemic dynamics are similar to those in data-limited countries) to train the prediction model and use the trained model to predict the number of cases and deaths in data-limited countries [32]. Kong et al. adopt similar transfer strategies, with an integration of other disease-related factors (e.g., demographic, geographic, and behavioral features) [33]. Li et al. implement the transfer learning by first training the model using data from source countries (data-rich regions) and then fine-tuning model parameters using data from data-limited regions (target countries) [34]. However, these methods focus on country-level prediction and thus are not suitable for fine-scale transmission modeling.

To address these challenges, we propose to uncover the fine-scale spatiotemporal transmission patterns of COVID-19, which we name “TransCode,” to capture transmission dynamics and predict future infection risks. To quantitatively characterize the TransCode, we first construct the mobility trajectories of confirmed cases using their visiting records. We then estimate the transmissibility of individual cases in different locations based on their temporal infectiousness distribution. Integrating the spatial and temporal information, we represent the TransCode via spatiotemporal transmission networks. Figure 1b (right) shows an example of such a transmission network in Hong Kong, China where the nodes and edges represent the districts and cross-district transmission intensities, respectively.

Further, we propose a deep transfer learning model to adapt the TransCode to achieve fine-scale transmission characterization and risk prediction in regions where fine-scale data are limited. First, we match the disease dynamics of source region (data-rich region) and those of target region (data-limited region) via temporal segmentation and clustering to determine the most appropriate period from the source region for TransCode adaptation. Second, we develop a deep transfer learning model with a source module and a target module. The source module extracts features from the spatiotemporal transmission networks of the selected period of the source region and feeds the extracted features into the target module. The TransCode of the target region is obtained by minimizing the objective functions (i.e., the prediction errors) of both modules of the deep transfer model. Finally, we provide a theoretical analysis to guarantee the performance of the developed model.

To validate the effectiveness of TransCode for representing fine-scale transmission dynamics and predicting disease transmission risks, we conduct extensive experiments on seven densely populated metropolises: New York City, San Francisco, Toronto, London, Berlin, Tokyo, and Hong Kong. The TransCode of Hong Kong, China derived from the spatial transmission information and temporal infectiousness distribution of individual cases reveals the transmission patterns (e.g., the imported and exported transmission intensities) at the district and constituency levels during different COVID-19 outbreak waves. The high-risk constituencies predicted using the TransCode correspond to areas where compulsory testing was implemented by the government and infection cases were detected, validating the usability of TransCode for risk prediction and potential case identification.

Finally, we evaluate the adaptability of the TransCode of Hong Kong, China to the other six metropolises, where fine-scale visiting records of individual cases are not available. By adapting the TransCode of Hong Kong, China to these cities, we find that, although the transmission dynamics of these cities appear heterogeneous, they share some intrinsically common patterns of human mobility and contact behaviors at the metapopulation level. As a result, the adapted TransCode reflects the fine-scale spatiotemporal transmission patterns in these data-limited metropolises. Moreover, the proposed deep transfer learning model that incorporates the adapted TransCode outperforms state-of-the-art machine learning methods in transmission risk prediction, further demonstrating the capacity of the TransCode to uncover spatiotemporal transmission patterns. Characterization of the fine-scale transmission patterns (e.g., transmission distributions and imported/exported transmission risks of different districts) using the TransCode will facilitate the development of tailor-made intervention strategies to effectively contain disease transmission in the targeted regions.

## Methods

### Data collection and processing

The following data were collected to investigate COVID-19 transmission patterns in Hong Kong, China and construct the TransCode. First, we collected the confirmed COVID-19 case data in all 18 districts from January 23, 2020 to February 14, 2021, from the resources provided by the Department of Health, Hong Kong SAR Government [35]. The onset date, report date, and the buildings that the confirmed case visited in the 14 days before the case confirmation date were recorded for each case. We collected the latitude and longitude of each building using the Google Geocoding API and identified the constituency that each building belongs to using the constituency area shapefile, with the latitudes and longitudes of the constituency boundaries included. We collected relevant data from the official websites of the other six metropolises: New York City [36], San Francisco [37], Toronto [38], London [39], Berlin [40], and Tokyo [41]. These data contained the daily case numbers at the city and district/borough levels. The spatial resolutions of these cities are provided in the Additional file 1.

### TransCode construction

**Fig. 2:**
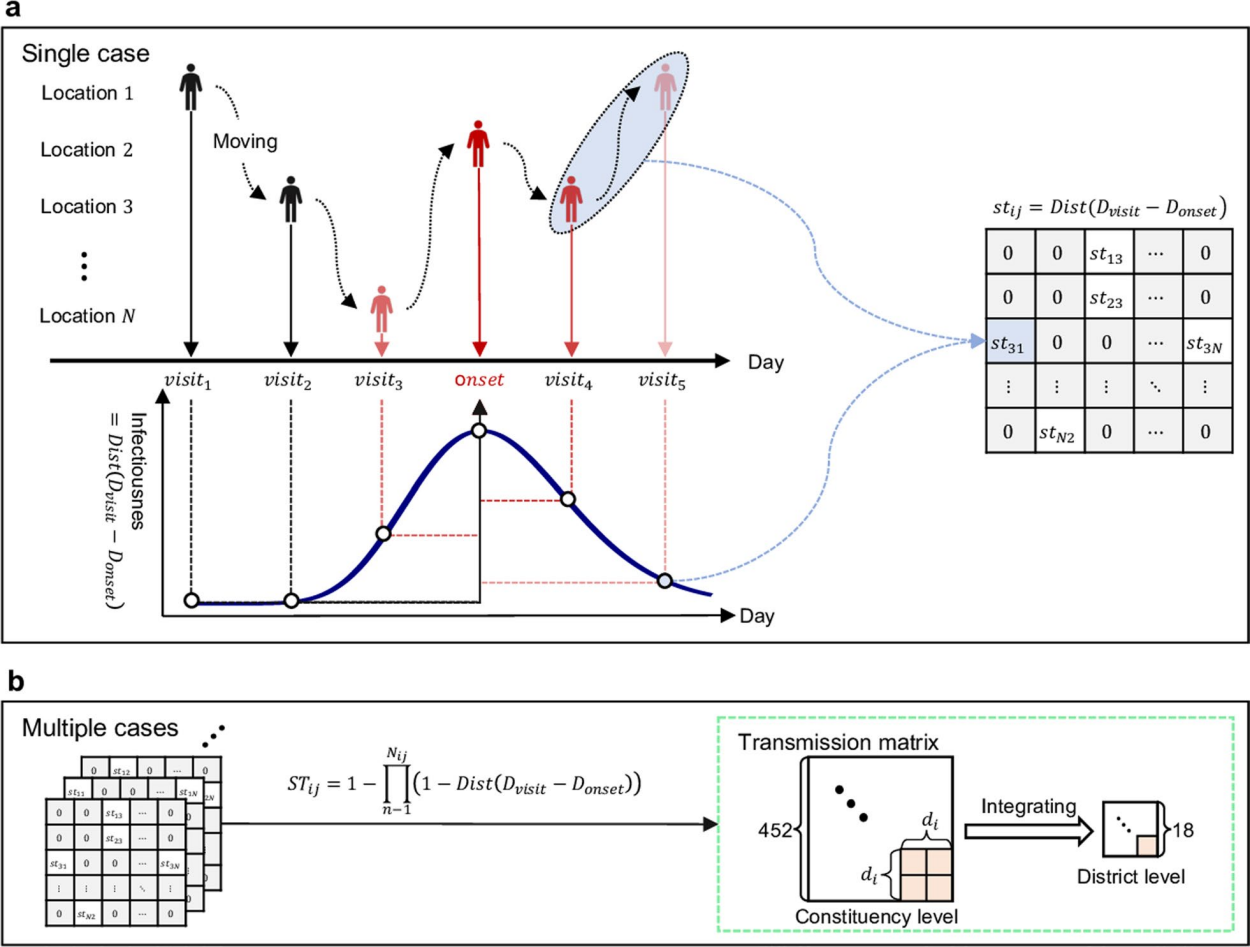
TransCode construction. **a** Calculating the transmissibility of a single case according to the individual’s visiting records (left). The closer the visiting date to the onset date, the higher the transmissibility. The case’s cross-constituency transmission matrix is obtained by calculating the transmissibility of the individual case for all constituency pairs (right). **b** The cross-constituency transmission matrix (i.e., the constituency-level TransCode) at the metapopulation level is formed by aggregating the transmission matrices of all individual cases (left). The cross-district transmission matrix (i.e., the district-level TransCode) is obtained by identifying the district that each constituency belongs to and summating all elements in the constituency-level matrix within that district block (right)

To characterize the transmission patterns at the metapopulation level, we start by measuring the transmissibility of a single case, which is assumed to follow the infectiousness distribution [51]. Specifically, for any two locations (i.e., constituencies) $p$ and $q$ in a case mobility trajectory (e.g., $p=3$ and $q=1$ on the top left of Fig. 2a) on the same day, assuming that constituency $p$ (corresponding to the fourth visit of the individual) was visited before constituency $q$ (corresponding to the fifth visit of the individual), we use the difference between the visit date and the onset date as the infectiousness distribution input (Fig. 2a, bottom left) to infer the individual case transmissibility from $p$ to $q$. By calculating the individual case transmissibility for all constituency pairs, we obtain the cross-constituency transmission network for that case (represented as a matrix with the size $N\times N$, where $N$ denotes the number of constituencies; Fig. 2a, right). Next, we aggregate the transmission matrices of all cases to form the cross-constituency transmission matrix (i.e., the constituency-level TransCode) at the metapopulation level (Fig. 2b, left). Finally, we construct the cross-district transmission matrix (i.e., the district-level TransCode) by identifying the district that each constituency belongs to and summating all elements in the constituency-level matrix within that district block (Fig. 2b, right). The detailed construction and calculation methods are introduced as follows.

### Transmissibility of individual cases

We construct the disease transmission networks in Hong Kong, China using the spatiotemporal information of individual cases. From the visiting records of confirmed cases, we calculate the disease transmissibility of a single case from constituency $i$ to $j$ using the following equation:

1 $$\begin{array}{c}{st}_{ij}=Dist({D}_{visit}-{D}_{onset}),\end{array}$$

where ${st}_{ij}$ represents the transmission intensity of one case from location $i$ to $j$; $Dist(\bullet )$ is the infectiousness distribution of confirmed cases in terms of the number of days before or after symptom onset, which is inferred using the temporal transmission patterns of 77 infector–infectee pairs [51]; ${D}_{onset}$ is the date of onset; and ${D}_{visit}$ is the visiting date from constituency $i$ to $j$. In asymptomatic cases, there are no explicit onset date records. Therefore, we use the serial interval distribution to describe infectiousness and set the report day as the onset day, defining the serial interval as the length of time between the symptom onset dates of consecutive cases in transmission chains. We use the difference between the visiting date and the onset date as the input for the distribution function to calculate the infectiousness of individual cases.

### Individual transmissibility aggregation for TransCode construction

For all confirmed cases, the total transmissibility from constituency $i$ to $j$ is calculated as follows:

2 $$\begin{array}{c}{ST}_{ij}=1-\prod_{n=1}^{{N}_{ij}}\left({1-Dist\left({D}_{visit}-{D}_{onset}\right)}_{n}\right),\end{array}$$

where ${N}_{ij}$ is the number of visits (contributed by all cases) from constituency $i$ to $j$. The ${ST}_{ij}$ is the ($i,j$)^th^ element of the constituency-level transmission matrix (network), i.e., the constituency-level TransCode. Hong Kong, China has 452 constituencies, so the size of the matrix is 452 × 452. We then summate all elements in the district block to obtain the 18 × 18 district-level transmission matrix. For instance, the district-level transmission intensity from district E (Yau Tsim Mong, with 20 constituencies) to H (Wong Tai Sin, with 25 constituencies) is represented by the summation of all elements in this 20 × 25 block.

### Transmission intensity prediction via TransCode

To validate the TransCode, we use a Gaussian process model [52] with deep kernel to infer the future transmission intensity of different constituencies and identify constituencies with potential transmission risk, i.e., those with high exported transmission intensity. For the prediction task, let $T\in {\mathbb{R}}^{1\times D}$ represent the exported transmission intensity of a constituency in $D$ consecutive days, which is a time series. We split the data into the training set, which comprises the first *L* days’ data (i.e., ${T}_{\left[1:L\right]}$), and the test set, which comprises the last $(D-L)$ days’ data (i.e., ${T}_{\left[L+1:D\right]}$). We then formulate the time series prediction as the following regression problem:

3 $$\begin{array}{c}{{\varvec{o}}}_{t}=f\left({{\varvec{w}}}_{t}\right)+\varepsilon , \varepsilon \sim N\left(0, {\sigma }^{2}\right),\end{array}$$

where $\varepsilon$ denotes the noise that follows the zero-mean normal distribution, ${\sigma }^{2}$ is the variance of the noise, $f\left(\bullet \right)$ is the mapping function, and ${{\varvec{w}}}_{t}{\in {\mathbb{R}}}^{1\times P}$ and ${{\varvec{o}}}_{t}{\in {\mathbb{R}}}^{1\times Q}$ are the input and output of the function $f\left(\bullet \right)$ for the time step $t$, respectively. In our case, ${{\varvec{w}}}_{t}$ represents the past $P$ days’ transmission intensity, and ${{\varvec{o}}}_{t}$ represents the next $Q$ days’ intensity that is to be predicted. In this study, we set $P=14$ and $Q=1$, i.e., we use the transmission intensity of the last two weeks to predict the intensity of the next day. For simplicity, we omit the subscript of ${{\varvec{w}}}_{t}$ and ${{\varvec{o}}}_{t}$ in the following description.

$f\left(\bullet \right)$ follows a Gaussian process, denoted as $f\left(\bullet \right)\sim GP(\mu \left(\bullet \right),K\left(\bullet \right))$, where $\mu \left(\bullet \right)$ and $K\left(\bullet \right)$ denote the mean function and the covariance function, respectively:

4 $$\begin{array}{c}\mu \left({\varvec{w}}\right)=E\left(f\left({\varvec{w}}\right)\right),\end{array}$$

5 $$\begin{array}{c}K\left({\varvec{w}}, {\varvec{w}}\right)=E\left(\left(f\left({\varvec{w}}\right)-\mu \left({\varvec{w}}\right)\right){\left(f\left({\varvec{w}}\right)-\mu \left({\varvec{w}}\right)\right)}^{T}\right).\end{array}$$

We follow the standard setting [45] to set $\mu \left({\varvec{w}}\right)=0$ and use the deep kernel ${k}_{deep}\left(\bullet \right)$ as the covariance function:

6 $$\begin{array}{c}{k}_{deep}\left({\varvec{w}}, {\varvec{w}} \right)=v\left({GRU({\varvec{w}})}^{T}GRU({\varvec{w}}))\right),\end{array}$$

where GRU denotes the gated recurrent unit [43], a deep structure for sequence modeling, and $v$ is the coefficient of the deep kernel, which is determined automatically during the model training procedure.

We denote the parameter set of the learning model as $\delta$. To infer the $\delta$ that best fits the training data, we aim to minimize the negative log marginalized likelihood (NLML) as follows:

7 $$\begin{array}{c}NLML=-\mathrm{ln}p\left({\varvec{o}}|GRU\left({\varvec{W}}\right), {\varvec{\delta}}\right)= \frac{1}{2}{{\varvec{o}}}^{T}{{\varvec{\Sigma}}}_{\delta }^{-1}o+\frac{1}{2}\mathrm{lndet}{{\varvec{\Sigma}}}_{\delta }+\frac{n}{2}\mathrm{ln}2\pi ,\end{array}$$

where ${{\varvec{\Sigma}}}_{\delta }={{\varvec{K}}}_{\delta }+{\sigma }^{2}{\varvec{I}}$, with ${{\varvec{K}}}_{\delta }$ being the covariance (kernel) matrix and ${\varvec{I}}$ being the identity matrix, and ${\varvec{W}}\in {\mathbb{R}}^{P\times (L-P-Q)}$ and ${\varvec{o}}\in {\mathbb{R}}^{(L-P-Q)\times Q}$ are constructed from the training set. The parameters of the deep structure and kernel function are jointly optimized using the above objective function. We denote the transmission intensity that we aim to predict as ${{\varvec{o}}}^{*}\in {\mathbb{R}}^{(D-L-P-Q)\times Q}$ and the corresponding historical intensity used for prediction as ${{\varvec{W}}}^{*}{\in {\mathbb{R}}}^{p\times (D-L-P-Q)}$. Then ${{\varvec{o}}}^{*}$ is predicted as follows:

8 $$\begin{array}{c}{{\varvec{o}}}^{*}={k\left(GRU({{\varvec{W}}}^{*}),GRU({\varvec{W}})\right)}^{T}{({{\varvec{K}}}_{\delta }+{\sigma }^{2}{\varvec{I}})}^{-1}o.\end{array}$$

### TransCode adaptation

To evaluate the usability of the TransCode, we conduct a comprehensive investigation on six representative metropolises with high population densities: New York City, San Francisco, Toronto, London, Berlin, and Tokyo. Because the fine-scale (district-level) trajectory/visiting records of infection cases are not available in these cities, it is difficult to directly construct spatiotemporal transmission networks. To address this challenge, we propose a deep transfer learning model to adapt the TransCode of Hong Kong, China to those cities. Specifically, we consider Hong Kong, China as the source city and the other six metropolises as target cities. Adapting the TransCode from the source city to the target cities involves two phases: (1) transmission labeling and (2) deep transfer learning.

**Fig. 3:**
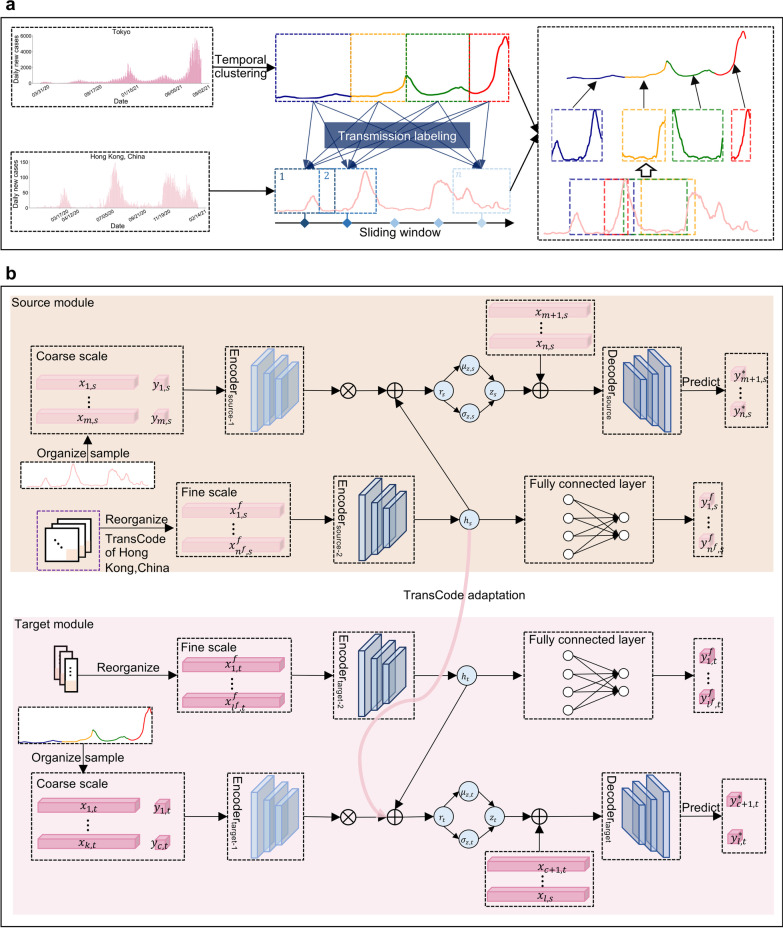
The TransCode adaptation procedure. **a** Transmission labeling. The city-level case dynamics of the target city and the source city (left). Partitioning of the target city case dynamics via temporal clustering and matching of the most similar segment pairs from the target city and source city (middle). The transmission labeling results (right). **b** The proposed deep transfer learning model. The source module of the model (top). The city-level case dynamics and the district-level TransCode of the source city are the input of the coarse-scale encoder and the fine-scale encoder, respectively. The fine-scale encoder extracts temporal features from the input and feeds the extracted features into the target module to infer the TransCode of the target city. The target module of the model (bottom). The city-level case dynamics and district-level case dynamics of the target city are the input of the coarse-scale encoder and the fine-scale encoder, respectively. By integrating the feature representations provided by the source module, the target module infers the target city TransCode and predicts the future transmission dynamics of the target city

For the transmission labeling phase, we first partition the city-level case dynamics of the target city into multiple temporal segments (i.e., periods) via temporal clustering. We then identify the source city segment most similar to each segment of the target city, allowing us to label the segments of the target city using the segments of the source city. The transmission labeling procedure is shown in Fig. 3a. After labeling the temporal segments of the target city, we use deep transfer learning to adapt the source city TransCode to the target city.

The deep transfer learning model is composed of a source module and a target module. First, the source module extracts features from the TransCode, i.e., the transmission network, of the selected temporal segment of the source city. Second, the features extracted from the source city TransCode are fed into the target module to generate the target city TransCode. By minimizing the objective functions (i.e., the prediction errors) of both modules of the deep transfer learning model, we expect that the target city TransCode can characterize the fine-scale transmission patterns, thus providing accurate predictions of transmission risks in the target city. The proposed deep transfer learning procedure for TransCode adaptation is illustrated in Fig. 3b; the technical details are provided in as follows. To guarantee the validity of the proposed model, we provide a theoretical analysis, discussing the conditions for TransCode adaptation. Please refer to the Additional file 1 for more information.

### Hierarchical aligned cluster analysis for transmission labeling

Before adapting the TransCode of the source location to the target location, we partition the city-level case sequence of the target location into several segments via temporal clustering (i.e., mean-shift clustering [46]) and label each segment using the most similar segment from the case sequence of the source city (Hong Kong, China). The selection is based on the hierarchical aligned cluster analysis [44]. Given ${\varvec{a}}=\left[{a}_{1},\dots {a}_{{n}_{a}}\right]\in {\mathbb{R}}^{1\times {n}_{a}}$ being the segment of the target city and ${\varvec{b}}=[{b}_{1},\dots {b}_{{n}_{b}}]\in {\mathbb{R}}^{1\times {n}_{b}}$ being the case sequence of the source city, the similarity function between ${\varvec{a}}$ and ${\varvec{b}}$ is defined as follows:

9 $$\begin{array}{c}\tau \left({\varvec{a}},{\varvec{b}}\right)=\frac{{u}_{{n}_{a}{n}_{b}}}{{n}_{a}+{n}_{b}}, {u}_{ij}=max\left\{\begin{array}{c}{u}_{i-1,j}+{k}_{ij}\\ {u}_{i-1,j-1}+{2k}_{ij}\\ {u}_{i,j-1}+{k}_{ij}\end{array}\right.,\end{array}$$

where ${\varvec{U}}\in {\mathbb{R}}^{{n}_{a}\times {n}_{b}}$ is the cumulative kernel matrix initialized from the upper left, i.e., ${u}_{\mathrm{1,1}}=2{k}_{\mathrm{1,1}}$, and ${k}_{ij}=\mathrm{exp}(-\frac{{\Vert {a}_{i}-{b}_{j}\Vert }^{2}}{2{\sigma }^{2}})$ is the radial basis function kernel. In our study, we set $\sigma =1$. The similarity is calculated between the segment of the target city and all subsequences of the source city’s case sequence. The subsequence of the source city with the highest similarity is selected as the label for the target city’s segment.

### Deep transfer learning model for TransCode adaptation

The proposed deep transfer learning model comprises a source module and a target module. Both modules have a two-layer structure to capture information from the coarse and fine scales, respectively. Specifically, the first layer in both modules captures dynamics at the coarse scale, i.e., the city level. The second layer in the source module and the target module extracts features from the source city (Hong Kong, China) TransCode and the fine-scale case trend of the target city, respectively.

In both modules, we use the neural process structure [47] in the first layer and the GRU structure in the second layer. We transfer the information extracted from the source module to the target module by incorporating the learned hidden representations of the source city’s disease transmission matrix and the feature representations of the target city’s fine-scale case trend so as to benefit the case prediction of the target city. In the first layer of the source module, the hidden representation is given as follows:

10 $$\begin{array}{c}{{\varvec{r}}}_{s}=\left(\sum_{i=1}^{m}{{\varvec{r}}}_{i,s}\right)\oplus {{\varvec{h}}}_{s}=\left(\sum_{i=1}^{m}{Encoder}_{source-1}\left({{\varvec{x}}}_{i,s},{y}_{i,s}\right)\right)\oplus {{\varvec{h}}}_{s},\end{array}$$

where $\sum_{i=1}^{m}{{\varvec{r}}}_{i,s}=\sum_{i=1}^{m}{Encoder}_{source-1}\left({{\varvec{x}}}_{i,s},{y}_{i,s}\right)$ is the aggregation (denoted as $\otimes$ in Fig. 3) of the encoder’s output of each context sample, the ${Encoder}_{source-1}$ denotes the multi-layer perceptron (MLP) with 5 layers, ${{\varvec{h}}}_{s}$ is the output of the ${Encoder}_{source-2}$ (1 layer GRU) in the second layer of the source module, and $\oplus$ is the concatenation operator (Fig. 3). The approximate posterior of ${{\varvec{z}}}_{s}$, denoted as $q\left({{\varvec{z}}}_{s}|\bullet \right)$, follows a normal distribution $N({{\varvec{\mu}}}_{{\varvec{z}},s},{{\varvec{\sigma}}}_{{\varvec{z}},s})$ and is parameterized by ${{\varvec{r}}}_{s}$. Here, the mean ${{\varvec{\mu}}}_{{\varvec{z}},s}$ is parameterized by a neural network ${NN}_{{\varvec{\mu}}}\left({{\varvec{r}}}_{s}\right)$, i.e., ${{\varvec{\mu}}}_{{\varvec{z}},s}={NN}_{{\varvec{\mu}}}\left({{\varvec{r}}}_{s}\right)$, with ${NN}_{{\varvec{\mu}}}\left(\bullet \right)$ being a single-layer perceptron without activation function, and the variance ${{\varvec{\sigma}}}_{{\varvec{z}},s}$ is parameterized by a neural network ${NN}_{{\varvec{\sigma}}}$, i.e., ${{\varvec{\sigma}}}_{{\varvec{z}},s}={NN}_{{\varvec{\sigma}}}\left({{\varvec{r}}}_{s}\right)$, with ${NN}_{{\varvec{\sigma}}}\left(\bullet \right)$ being another single-layer perceptron without activation function. The ${{\varvec{z}}}_{s}$ is the hidden state that is concatenated with ${\left\{{{\varvec{x}}}_{i,s}\right\}}_{i=m+1,..,n}$ for the prediction of ${\left\{{y}_{i,s}\right\}}_{i=m+1,..,n}$. The target module structure is similar to that of the source module. In the first layer of the target module, the feature representations extracted from the source city TransCode are integrated with representations of the target city disease transmission: ${{\varvec{r}}}_{t}={Encoder}_{target-1}\left({{\varvec{x}}}_{i,t},{y}_{i,t}\right)\oplus {{\varvec{h}}}_{t}\oplus {{\varvec{h}}}_{s}$. Here, ${{\varvec{h}}}_{t}$ is the ${Encoder}_{target-2}$ (1 layer GRU) output in the second layer of the target module and is transformed into the shape of $({d}_{days}\times {d}_{district})\times {d}_{district}$, where ${d}_{days}$ is the number of days of data used for training, and ${d}_{district}$ is the number of districts in the target city.

Four categories of data are used as the deep transfer learning model input. We distinguish the categories using subscripts: (1) For the case trends in the source city at the coarse scale, we organize the data as input and output pairs for the model training and test. For the case trend data, we use a sliding window ${d}_{window}$ (which is set to 14 days in our study) to generate a set of samples${\left\{({{\varvec{x}}}_{i,s},{y}_{i,s})\right\}}_{i=1,\dots ,n}$, where ${{\varvec{x}}}_{i,s}$ is a ${d}_{window}$-dimensional vector that contains the case number (averaged) in consecutive ${d}_{window}$ days, ${y}_{i,s}$ is the case number on the following day, and $n$ is the number of sample pairs. (2) For the fine-scale TransCode in the source city, we treat the temporal sequence of each element in the matrix as a time series and generate a set of samples for those time series using the sliding window introduced before. We aggregate the samples for all elements as the set denoted as ${\left\{({{\varvec{x}}}_{i,s}^{f},{y}_{i,s}^{f})\right\}}_{i=1,\dots ,{n}^{f}}$, where ${n}^{f}$ is the number of sample pairs. (3) For the case trends in the target city at the coarse scale, the sample set with $l$ sample pairs, denoted as${\left\{({{\varvec{x}}}_{i,t},{y}_{i,t})\right\}}_{i=1,\dots ,l}$, is generated using the sliding window. (4) For the case trends in the target city at the fine scale, the sample set ${\left\{({{\varvec{x}}}_{i,t}^{f},{y}_{i,t}^{f})\right\}}_{i=1,\dots ,{l}^{f}}$ with ${l}^{f}$ sample pairs is generated using the sliding window on all of the districts’ case trends.

According to the training requirement of the neural process structure, the city-level samples are split into two sets: the context sample set and the target sample set. Using the coarse-scale case trends of the source city as an example, we use the first $m$ samples as the context samples and the remaining samples as the target samples, i.e., the context samples are denoted as ${\left\{({{\varvec{x}}}_{i,s},{y}_{i,s})\right\}}_{i=1,\dots ,m} (m<n$), and the target samples are denoted as ${\left\{({{\varvec{x}}}_{i,s},{y}_{i,s})\right\}}_{i=m+1,\dots ,n}$. We aim to predict ${\left\{{y}_{i,s}\right\}}_{i=m+1,..,n}$, denoted as ${\left\{{y}_{i,s}^{*}\right\}}_{i=m+1,..,n}$, by giving the input of the target samples ${\left\{{{\varvec{x}}}_{i,s}\right\}}_{i=m+1,..,n}$ and the context samples ${\left\{({{\varvec{x}}}_{i,s},{y}_{i,s})\right\}}_{i=1,\dots ,m}$. Similarly, for the target city, we use the first $c$ samples as the context samples and the remaining samples as the target samples.

The overall objective function of the developed deep transfer learning model for TransCode adaptation is as follows:

11 $${{\mathbb{E}}_{q\left({{\varvec{z}}}_{t}|{{\varvec{x}}}_{t},{y}_{t}\right)}}^{Target}\left[{\sum }_{i=m+1}^{n}\mathrm{log}p\left({y}_{i,t}^{*}|{{\varvec{z}}}_{t},{{\varvec{x}}}_{i,t}^{*}\right)+log\frac{q\left({{\varvec{z}}}_{t}|{\left\{\left({{\varvec{x}}}_{i,t},{y}_{i,t}\right)\right\}}_{i=1,\dots ,c}\right)}{q\left({{\varvec{z}}}_{t}|{\left\{\left({{\varvec{x}}}_{i,t},{y}_{i,t}\right)\right\}}_{i=1,\dots ,l}\right)}\right]+{\Vert {{\varvec{y}}}_{t}^{f}-{{\varvec{y}}}_{t}^{{f}^{*}}\Vert }_{Target}^{2}+ {{\mathbb{E}}_{q\left({{\varvec{z}}}_{s}|{{\varvec{x}}}_{s},{y}_{s}\right)}}^{Source}\left[{\sum }_{i=m+1}^{n}\mathrm{log}p\left({y}_{i,s}^{*}|{{\varvec{z}}}_{s},{{\varvec{x}}}_{i,s}^{*}\right)+log\frac{q\left({{\varvec{z}}}_{s}|{\left\{\left({{\varvec{x}}}_{i,s},{y}_{i,s}\right)\right\}}_{i=1,\dots ,m}\right)}{q\left({{\varvec{z}}}_{s}|{\left\{\left({{\varvec{x}}}_{i,s},{y}_{i,s}\right)\right\}}_{i=1,\dots ,n}\right)}\right]+{\Vert {{\varvec{y}}}_{s}^{f}-{{\varvec{y}}}_{s}^{{f}^{*}}\Vert }_{Source}^{2},$$

where ${{\varvec{y}}}_{t}^{{f}^{*}}$ and ${{\varvec{y}}}_{s}^{{f}^{*}}$ are the model predictions of ${{\varvec{y}}}_{t}^{f}{=\left\{{y}_{i,t}^{f}\right\}}_{i=1,\dots ,{n}^{f}}$ and ${{\varvec{y}}}_{s}^{f}{=\left\{{y}_{i,s}^{f}\right\}}_{i=1,\dots ,{l}^{f}}$, respectively. For both modules, the first term is the loss of the neural process structure (i.e., the evidence lower bound [47]), and the second term is the mean squared error loss of the GRU structure. We use the Adam optimizer and set the learning rate to 0.0006. The rectified linear activation function (ReLU) is used for the model.

We conduct one-step-ahead predictions for durations of one week and two weeks. Finally, we calculate the $MAE=\frac{\Vert {{\varvec{y}}}_{t}^{{f}^{*}}-{{\varvec{y}}}_{t}^{f}\Vert }{{l}_{test}}$ and $RMAE=\frac{\Vert {{\varvec{y}}}_{t}^{{f}^{*}}-{{\varvec{y}}}_{t}^{f}\Vert }{\Vert {{\varvec{y}}}_{t}^{f}\Vert }$ between the predicted value and the ground truth to evaluate the prediction accuracy.

## Results

In this section, we present the TransCode development and adaptation results. First, we introduce the process of developing the TransCode, present the TransCode of Hong Kong, China at both the district and constituency levels, and analyze the efficacy of the TransCode for identifying high transmission risk locations. Then, we describe the TransCode adaptation procedure, illustrate and discuss the adaptation of the TransCode of Hong Kong, China to the other six metropolises, and finally, show the transmission risk prediction efficacy of the proposed deep transfer learning model with the adapted TransCode.

### Developing the TransCode of Hong Kong, China

Four COVID-19 outbreak waves occurred in Hong Kong, China from January 23, 2020 to February 14, 2021 [2]: the first wave from late January to mid-February 2020; the second wave from March 17 to April 12, 2020; the third wave from July 5 to September 21, 2020; and the fourth wave from November 19, 2020 to February 14, 2021. As the duration, spatial scale, and infection peak of the first wave were much smaller than those of the later waves, we do not consider this wave in our study, i.e., the TransCode is based on the second, the third, and the fourth wave data.

**Fig. 4:**
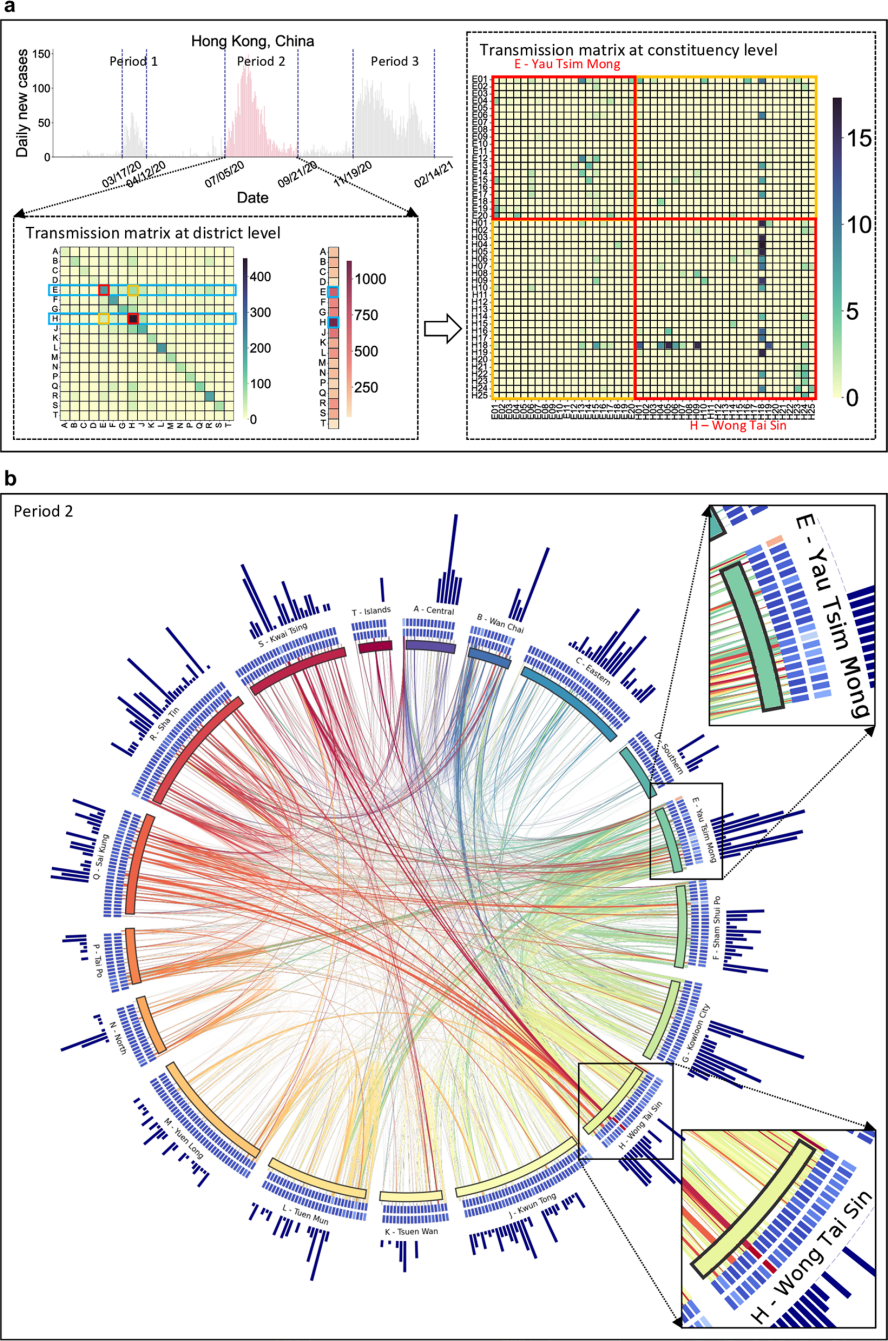
TransCode of the third wave (period 2) of COVID-19 outbreaks in Hong Kong, China. **a** The daily case numbers from January 23, 2020, to February 14, 2021, in Hong Kong, China (top left); period 2 is highlighted in pink. The district-level TransCode (i.e., the transmission network, which is shown as matrix) of period 2 (bottom left). The constituency-level TransCode of two representative districts [Yau Tsim Mong (E) and Wong Tai Sin (H)] (right). Darker colors on the matrices indicate higher case numbers within the district/constituency and higher transmission intensities between the districts/constituencies, respectively. **b** Chord diagram of the TransCode at the constituency level. The outermost bar charts show the cumulative case numbers in the 452 constituencies. Next to the bar charts, the two-layer heatmaps illustrate the exported (outside layer) and imported (inside layer) disease transmission intensities of each constituency, respectively. Red indicates high transmission intensity, and blue indicates low transmission intensity. Next to the heatmaps, the inner circle of the chord diagram is divided into 18 pieces, each a different color, representing the districts. The innermost connections show the disease transmission between different constituencies. The color of the edge shows the district to which the constituency belongs, and the width of the edge denotes the transmission intensity. Yau Tsim Mong (E) and Wong Tai Sin (H), two representative districts, are enlarged on the top right and bottom right, respectively

We illustrate the TransCode of the third COVID-19 outbreak wave (period 2: July 5 to September 21, 2020) in Hong Kong, China at the district and constituency levels in Fig. 4. The cross-district transmission network is shown as a matrix on the bottom left of Fig. 4a, and the constituency-level transmission network (shown as matrix on the right of Fig. 4a) between two densely populated districts with the highest cumulative case numbers, Yau Tsim Mong (administrative code E) and Wong Tai Sin (administrative code H), is on the right. At both spatial levels, the case number and the transmission intensity are unevenly distributed on different districts, and edges of the transmission network (non-diagonal elements of the matrix), respectively, which are indicated by different color intensities. This observation demonstrates the spatial heterogeneity of disease transmission, validating the necessity of characterizing such fine-scale information to capture transmission dynamics.

Next, we create a chord diagram (Fig. 4b) to visualize the TransCode transmission dynamic details at both levels. The districts with relatively high transmission intensity in the TransCode are Yau Tsim Mong (E), Sham Shui Po (F), Kowloon City (G), Wong Tai Sin (H), Kwun Tong (J), and Sha Tin (R), consistent with the characteristics of these districts. For example, Kowloon City (G) includes many densely distributed old infrastructure/buildings, which present high infection risks to residents due to poor ventilation and old sewerage system. Furthermore, traditional Chinese tea restaurants (Cha Chaan Teng), in which people tend to stay and chat with each other for long periods without masks (morning/afternoon tea), are widespread in this district, representing a hotspot for disease transmission. Sham Shui Po (F) is the district with the lowest average household income and the highest percentage of older adults; several families often share a single small apartment, creating a high-risk environment for household transmission. In addition to the high transmission intensity, the TransCode shows that these districts also have more cross-district interactions with each other than with the remaining districts, matching well with the characteristics of these districts. For example, Yau Tsim Mong (E) is a district with many shopping malls, tourist attractions, clubs, and restaurants for commercial, entertainment, and travel activities. Yau Tsim Mong (E) is also the main transportation hub in Hong Kong, China, providing ample opportunity for cross-district mobility and social gathering behaviors and enabling widespread COVID-19 transmission to and from this district.

To dive into the fine-scale (constituency-level) transmission patterns, we select Yau Tsim Mong (E) and Wong Tai Sin (H) for further analysis. Although the case numbers in both districts are very high, their constituency-level transmission patterns differ. In Yau Tsim Mong (E), most constituencies have high imported and exported transmission intensity. As shown in the two-layer heatmap in Fig. 4b, the constituencies with the highest exported transmission intensity are E01 (Tsim Sha Tsui West), E06 (Mong Kok West), E12 (Tai Nan), E13 (Mong Kok North), E14 (Mong Kok East), E15 (Mong Kok South), E17 (East Tsim Sha Tsui & King’s Park), and E20 (Tsim Sha Tsui Central); the constituencies with the highest imported transmission intensity are E01, E13, and E15. The reason is that these constituencies have extremely high population densities and contain many social gathering venues, creating an opportune environment for close contact and COVID-19 transmission.

In contrast to the wide distribution of constituencies with high-intensity transmission in Yau Tsim Mong (E), the district of Wong Tai Sin (H) has more concentrated patterns. Specifically, a single constituency, H18 (Ching On), has much higher imported and exported transmission intensities than the other constituencies in this district, indicating spatially concentrated transmission during this period. This pattern is consistent with the real situation: several outbreaks occurred in the Tsz Wan Shan Shopping Centre, located in H18, from July 14 to July 27, 2020, accounting for the high intensity on the corresponding edges of the constituency-level transmission network. By characterizing such fine-scale transmission patterns, the subtle variations overlooked in coarse-scale overall case number reports can be captured, which is essential for accurately inferring the disease dynamics.

**Fig. 5:**
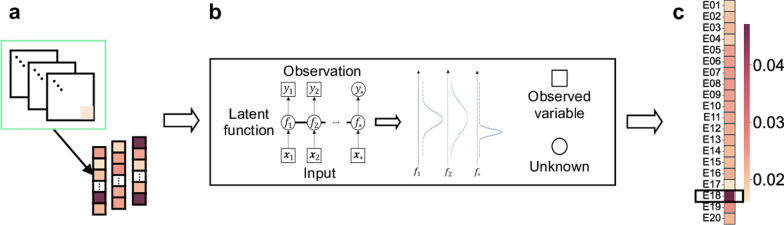
TransCode validation. **a** Data processing procedure. Each row of the constituency-level TransCode matrix of a district is summated to form the exported transmission intensity of the corresponding constituency. **b** Gaussian process regression with deep kernel. The model uses the historical exported transmission intensity as the input and infers the future transmission risk of each constituency. **c** Transmission risk prediction results for the district Yau Tsim Mong (E). Darker colors indicate a higher predicted transmission intensity; the constituency Jordan North (E18) has the highest predicted risk

To validate the TransCode of Hong Kong, China, we use it and a computational method to identify the constituencies where invisible transmission may exist. Invisible transmission, which is generally caused by unreported cases, is very difficult to detect. In our evaluation, we first quantify the daily transmission risk of a constituency by summating the daily transmission intensities exported from this constituency to all constituencies, including itself (i.e., conducting the row summation in the constituency-level TransCode matrix; Fig. 5a), to obtain a time series of the daily transmission risk for each constituency. Next, we adopt a Gaussian process regression [42] with deep kernel, a representative machine learning model, to predict the future transmission risk of each constituency (Fig. 5b). A higher predicted transmission risk indicates a greater probability of invisible transmission. The details of the adopted method are reported in “Transmission intensity prediction via TransCode” section.

We use the period 3 data for training and testing. Specifically, we use the TransCode from November 19, 2020, to January 17, 2021 to train the Gaussian process regression model and predict the exported transmission intensity from January 18 to January 24, 2021 using the trained model. We average the predicted intensities of each constituency in these seven days to obtain the predicted risk for the corresponding constituency. We show the prediction results for the Yau Tsim Mong (E) district in Fig. 5c. Among the 20 constituencies in this district, Jordan North (E18) has the highest predicted transmission risk, which is consistent with the fact that on January 23, 2021, compulsory testing was implemented in this constituency, and 13 cases were detected [56, 57]. This number is much higher than the number of cases identified in other constituencies during the same week, confirming that the TransCode accurately captured the disease transmission patterns.

### TransCode adaptation to data-limited metropolises

**Fig. 6:**
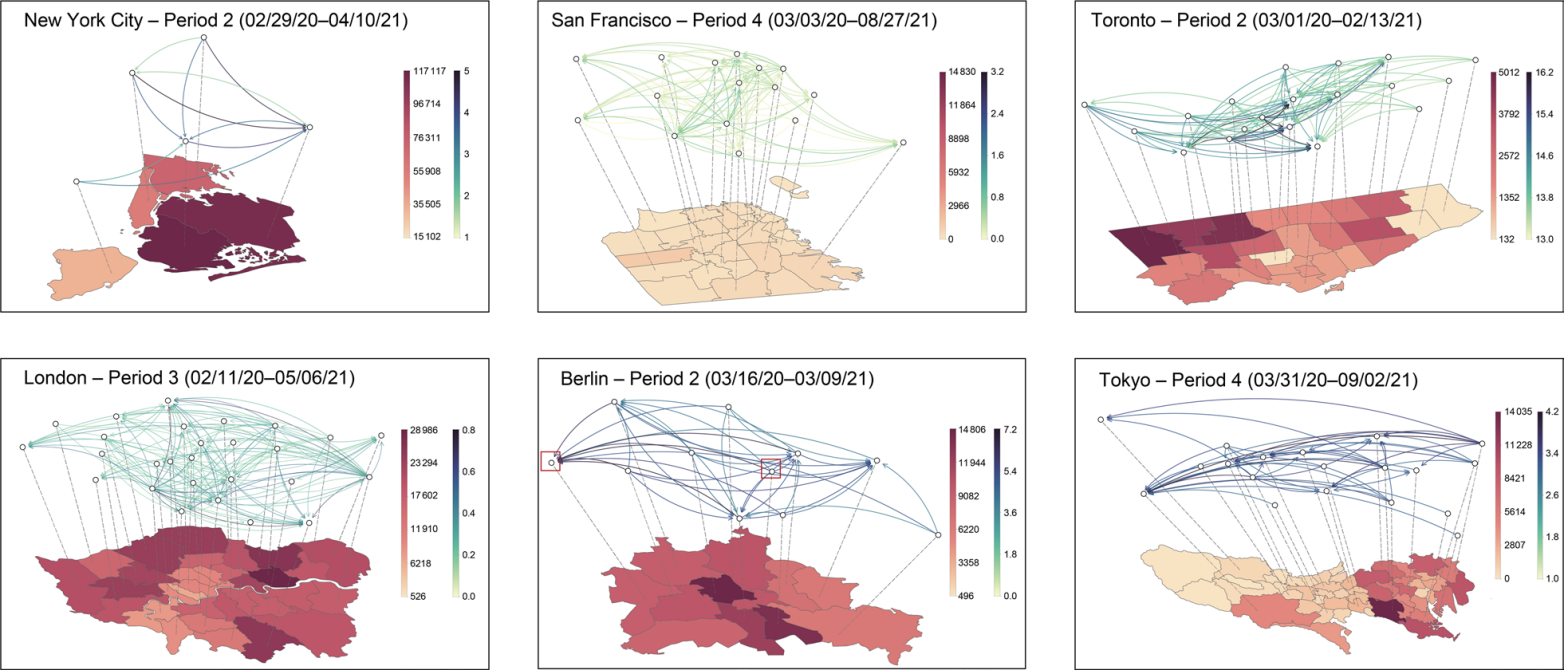
The TransCodes adapted for New York City, San Francisco, Toronto, London, Berlin, and Tokyo during different periods. In each subfigure, the map shows the number of cumulative confirmed cases in each district during the corresponding period (darker colors indicate higher case numbers); the transmission network represents the uncovered or inferred TransCode. The network nodes correspond to the districts, and the directed network edges show transmission from one district to another (darker edge colors indicate higher transmission intensities)

We show the district-level TransCodes (transmission networks) of six data-limited metropolises for the selected periods in Fig. 6. More comprehensive TransCode results of these six cities for all periods are provided in Additional file 1: Figs. S3–S8. We show the heterogeneity of disease transmission patterns between cities. For example, Berlin and Tokyo have similar cumulative case numbers, and their case maps show that the confirmed cases are mainly distributed in regions with large populations, such as Mitte and Neukölln in Berlin and the metropolitan area in Tokyo. However, the TransCodes in these two cities demonstrate different transmission patterns at the district level: disease propagation is concentrated in the eastern area of Tokyo but is relatively evenly distributed in Berlin. This observation from the TransCode is readily explainable: the eastern area (metropolitan area) is the commercial and economic center of Tokyo, and transportation between eastern and western Tokyo is not convenient, e.g., it takes more than two and half hours (by train and subway) to travel from Edogawa City (located near the eastern border of Tokyo) to Okutama Town (located near the western border of Tokyo). Therefore, disease transmission in Tokyo tends to be concentrated in the eastern area. In Berlin, Friedrichshain-Kreuzberg (marked by the red box in the center of the Berlin subfigure of Fig. 6) is the transmission hub, located in the center of Berlin and easily accessible from other districts (i.e., less than one hour of travel), facilitating the relatively easy spread of COVID-19 throughout Berlin.

Further, we can uncover more fine-scale transmission pattern details in these two cities by examining their TransCodes. Friedrichshain-Kreuzberg has the highest exported transmission risk in Berlin, which is not surprising because this district has the highest population density of the 12 districts [61]. Moreover, Friedrichshain-Kreuzberg has many inexpensive apartments and a lot of young residents; the many bars, cafes, and nightclubs lead to intensive social contacts for potential COVID-19 spread. Meanwhile, Spandau (marked by the red box on the left of the Berlin subfigure of Fig. 6), located in the west of Berlin, has the smallest population but the highest imported transmission risk. This high transmission risk can be ascribed to the well-developed modern industries, such as chemical and electrical factories, that bring many people into this district for work, thus increasing the imported transmission risk in this district.

**Fig. 7:**
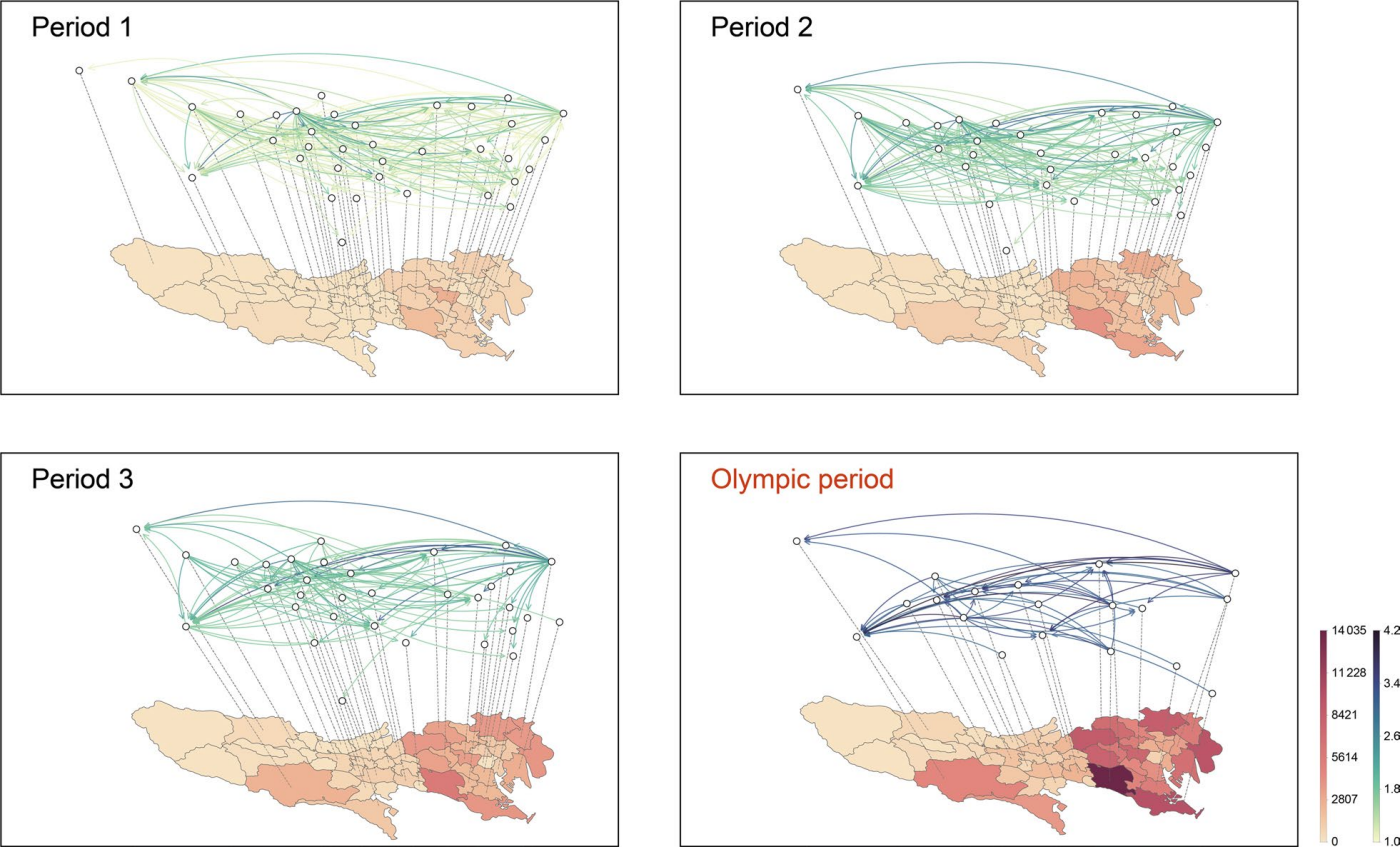
Results of temporal clustering of the case dynamics and the inferred TransCode for each period in Tokyo. **a** The sequence is partitioned into four periods, demonstrating different temporal patterns. The last segment is called the Olympic period because the Tokyo Summer Olympics were held during this period (July 23 to August 8, 2021), representing the main cause of this outbreak wave. **b** Visualization of the inferred TransCodes for four periods in Tokyo. In each subfigure, the map shows the number of cumulative confirmed cases in each district during the corresponding period (darker colors indicate higher case numbers); the transmission network represents the inferred TransCode. The network nodes correspond to the districts, and the directed network edges show transmission from one district to another (darker edge colors indicate higher transmission intensities)

In the inferred Tokyo TransCode, we can observe clear temporal heterogeneity in addition to spatial heterogeneity between the eastern and western areas. We show the temporal clustering results of the Tokyo case dynamics in Fig. 7a. The technique that generates the temporal clustering results was described in “Hierarchical aligned cluster analysis for transmission labeling” section. The sequence is segmented into four periods, demonstrating different temporal patterns. Period 4, which corresponds to the Tokyo Summer Olympics (July 23 to August 8, 2021), shows much higher COVID-19 spread (in terms of the daily infection number) than periods 1–3. This observation is consistent with the results in other studies [62–65]. Lau et al. estimated that different countries, during the Olympics period, would bring the imported risk to Tokyo [62], with the USA and UK having the highest probability for risk importation. A simulation study conducted by Yoneoka et al. showed that the number of cases per million population on the final day of the Games would be more than double that in the hypothetical situation of the Olympics Games not being held in Japan [63]. Jung et al. and Zhu et al. assessed the risk by taking the effectiveness of vaccination into consideration and more restricted strategies are suggested [64, 65]. The district-level TransCodes of these four periods in Tokyo, inferred by our deep transfer learning model are shown in Fig. 7b. The results are consistent with the city-level case dynamics: the transmission intensity of the TransCode during the Olympic period (period 4, bottom right subfigure) is much higher than that during the other three periods. Specifically, the trend of case concentration in the eastern area is more obvious during the Olympic period than during periods 1–3. A possible reason is that ten competition venues for the Tokyo Olympics are located in this metropolitan area, attracting many local residents and foreign tourists to celebrate this international sports event and thus intensifying disease transmission in this area during the Olympic period.

In addition to inferring the TransCodes of the six metropolises by adapting the TransCode of Hong Kong, China, we also validate their effectiveness for predicting disease dynamics (in terms of case numbers) at the district level. We compare our TransCode-enabled deep transfer learning model with four representative approaches designed for disease risk prediction or time series prediction: (1) the Susceptible-Exposed-Infectious-Removed (SEIR) model [58], an ODE-based method for disease dynamics modeling; (2) the Mortality Risk Prediction Model for COVID-19 (MRPMC) [60], an ensemble machine learning model for disease prediction; (3) a recent deep transfer learning model based on the long short-term memory (LSTM) network specifically designed for COVID-19 case trend prediction [31] (LSTM-T); and (4) the Informer [59], a state-of-the-art deep learning model for time series prediction. We evaluate the results of the five methods (including our method) on two prediction tasks: one-week-ahead (7 days) prediction, and two-weeks-ahead (14 days) prediction. The mean absolute error (MAE) and the relative MAE (RMAE) are used as evaluation metrics.

**Fig. 8:**
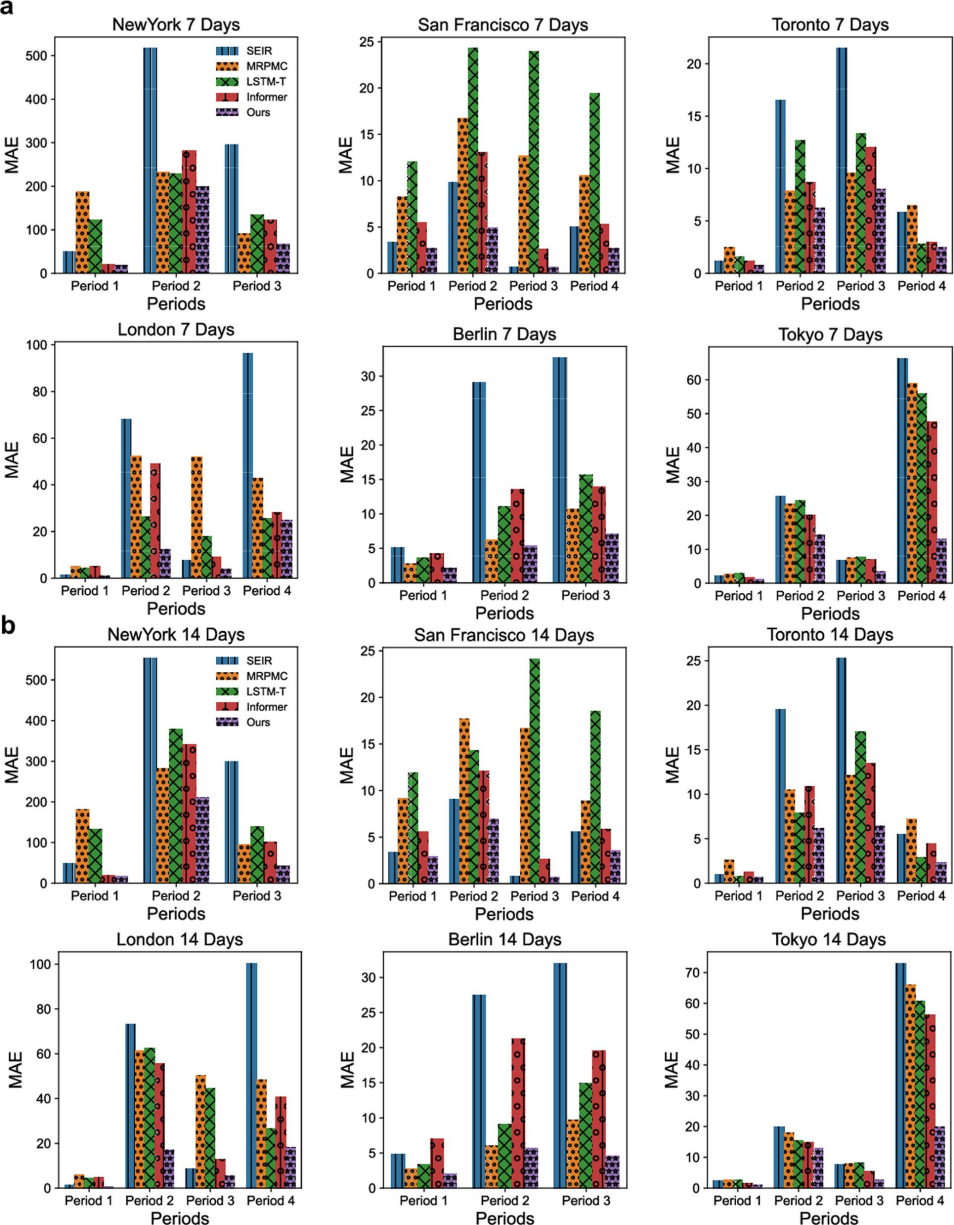
MAE of the district-level case number predictions of SEIR, MRPMC, LSTM-T, Informer, and our method in New York City, San Francisco, Toronto, London, Berlin, and Tokyo. **a** The MAE of one-week-ahead (7 days) predictions. **b** The MAE of two-weeks-ahead (14 days) predictions. Lower MAE values indicate better prediction performance. MAE: mean absolute error; SEIR: the Susceptible-Exposed-Infectious-Removed model [58]; MRPMC: Mortality Risk Prediction Model for COVID-19 [60]; LSTM-T: a long short-term memory network deep transfer learning model for COVID-19 case trend prediction [31]; Informer: a state-of-the-art deep learning model for time series prediction [59]; and Our method: a deep transfer learning model with adapted TransCodes

**Fig. 9:**
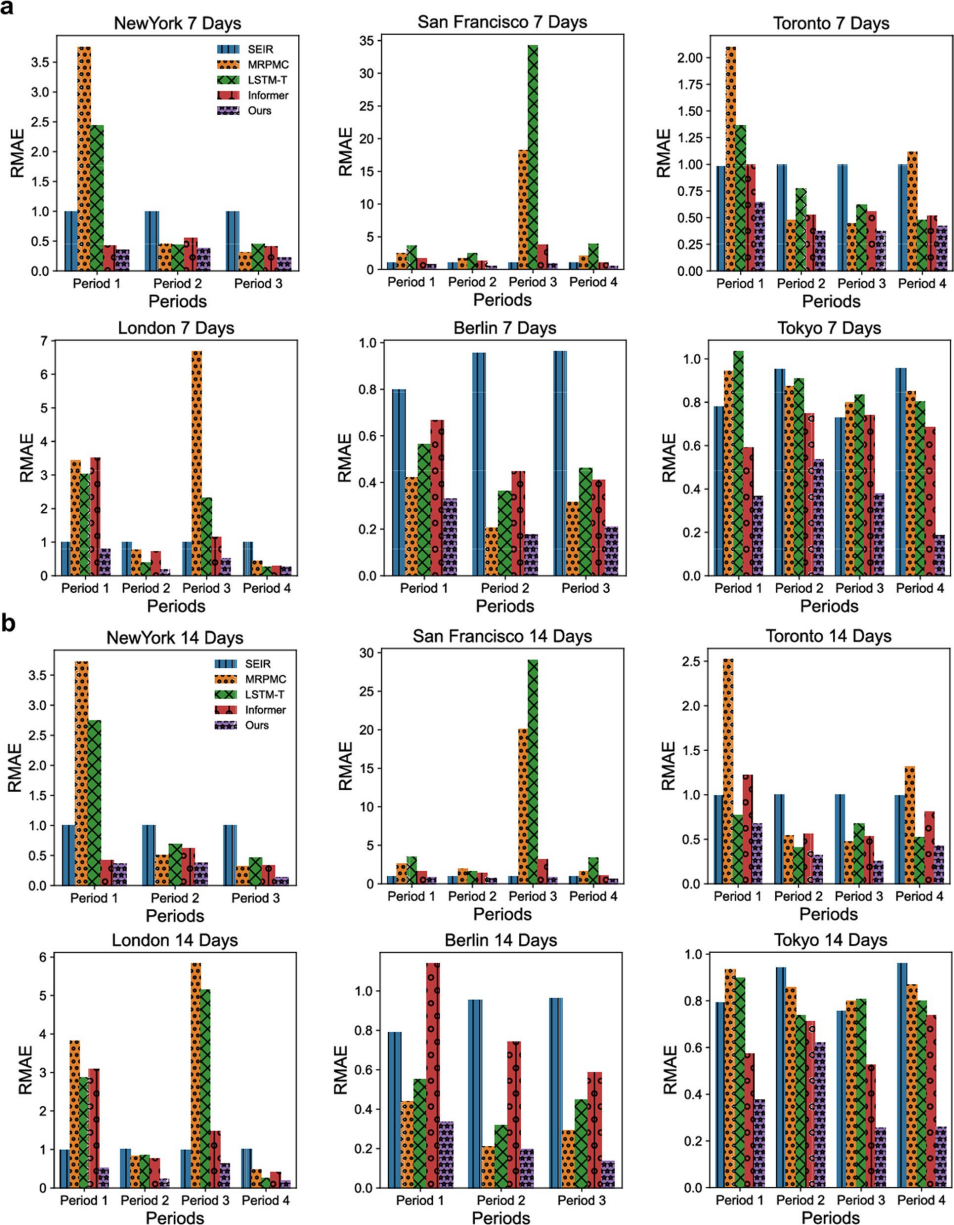
RMAE of the district-level case number predictions of SEIR, MRPMC, LSTM-T, Informer, and our method in New York City, San Francisco, Toronto, London, Berlin, and Tokyo. **a** The RMAE of one-week-ahead (7 days) predictions. **b** The RMAE of two-weeks-ahead (14 days) predictions. Lower RMAE values indicate better prediction performance. RMAE: relative mean absolute error; SEIR: the Susceptible-Exposed-Infectious-Removed model [58]; MRPMC: Mortality Risk Prediction Model for COVID-19 [60]; LSTM-T: a long short-term memory network deep transfer learning model for COVID-19 case trend prediction [31]; Informer: a state-of-the-art deep learning model for time series prediction [59]; and Our method: a deep transfer learning model with adapted TransCodes

The prediction results of the five methods in six metropolises are shown in Fig. 8 (MAE) and Fig. 9 (RMAE). The performance of the SEIR model is not satisfactory, possibly because the real dynamics of disease transmission are too complicated to be fully captured by ODEs. The performance of LSTM-T is not robust when the number of infected cases is low, e.g., period 1 in New York City and periods 3 in San Francisco and London. The LSTM-T transfers the city-level case dynamics of all three periods rather than the fine-scale spatiotemporal transmission networks of the most appropriate segment; thus, the transferred information does not represent the disease transmission characteristics of the target cities well. The MRPMC integrates several basic learning models, namely a linear regression, a support vector machine, a gradient boost decision tree, and a multi-layer perceptron, for ensemble learning. However, these basic models are insufficient for modeling sophisticated nonlinear data, and thus their ensemble may not be powerful enough to characterize the complex spatiotemporal dynamics of COVID-19 transmission. The Informer, which boasts a powerful deep learning structure, achieves relatively good performance. However, the Informer only relies on target city data and does not exploit the useful information in the source city TransCode. By exploring the fine-scale spatiotemporal transmission patterns in the source city and adapting the TransCode to the target cities using a deep transfer architecture, our method achieves the best performance among the five methods in all scenarios, demonstrating that the TransCode effectively captures transmission dynamics and predicts future risks. More details of the experimental results are provided in the Additional file 1.

## Discussion

Coarse-scale case dynamics may not be sufficient to support the implementation of tailor-made interventions because they do not reflect complex transmission patterns. Locations (e.g., cities or districts) with similar cumulative case numbers can have heterogeneous fine-scale transmission patterns that require different intervention strategies. For instance, the Yau Tsim Mong (E) district and the Wong Tai Sin (H) district have high confirmed case numbers during the period from July 5 to September 21, 2020. However, the constituency-level transmission patterns of these two districts differ: transmission is evenly distributed in Yau Tsim Mong, whereas Wong Tai Sin has more concentrated outbreaks in a specific constituency. Thus, constituency-specific transmission patterns should be considered when planning intervention measures. For example, more general policies, such as mandatory masking and social distancing, could be implemented for the entire population of Yau Tsim Mong, while specific strategies, such as small-scale compulsory testing and quarantines, could be adopted in the Wong Tai Sin constituencies with high transmission risks.

In addition to transmission distribution patterns, the TransCode unveils the details of disease spread patterns, such as the imported and exported transmission risks, which can inform public health decision-making. For locations with high imported transmission risk, such as Hachioji in Tokyo, intervention measures, such as body temperature testing, Vaccine Passes (i.e., proof of vaccination), and rapid antigen testing, could be implemented to prevent disease importation. For places with high exported transmission risk, such as Setagaya during the Olympic period, policies to prohibit large-scale social gatherings could be immediately implemented to limit local outbreaks and disease exportation.

Building upon the TransCode developed for Hong Kong, China, the success of TransCode adaptation illustrates the potential for mining fine-scale disease transmission patterns in locations with limited data availability. The TransCode adaptations reveal some intrinsic commonalities between the transmission patterns in densely populated regions, even when the disease dynamics of these regions appear heterogeneous on a coarse scale. We use a deep transfer learning model to adapt the TransCode developed for data-rich regions to infer the fine-scale transmission patterns of COVID-19 in targeted data-limited regions. The successful adaptation of the TransCode of Hong Kong, China to six other metropolises in this study demonstrates the potential for TransCode application in other densely populated regions.

### Limitation of the study

The TransCode and the deep transfer learning model developed in this paper are useful and generalizable for uncovering fine-scale disease transmission patterns, and we demonstrated their effectiveness in seven representative metropolises on three continents. However, when applying the TransCode to other regions, region-specific factors, such as local human mobility and social contact patterns, should be taken into consideration to achieve accurate characterization of transmission patterns.

Moreover, as empirically shown and theoretically guaranteed, TransCode adaptation can be effective even when the coarse-scale disease dynamics of the source region and target region differ. However, the premise of TransCode adaptation success is the intrinsically common or similar transmission-triggering factors shared by the source and target regions. For example, Hong Kong, China and London, two metropolises investigated in this study, have similar population densities and well-developed public transportation systems. Moreover, both cities are global financial centers. As a result, the human mobility and contact behaviors and social activity patterns in these two cities are analogous, ensuring the effectiveness of the TransCode adaptation. Thus, similarities and differences between the source and target regions should be taken into account when adapting the TransCode to data-limited regions.

## Conclusions

To tackle the challenging issue of characterizing the fine-scale spatiotemporal transmission patterns of COVID-19 in densely populated regions, we propose the notion of TransCode and adapt it via a novel deep transfer learning model. The step-by-step demonstration and analysis in the Results Section show that the TransCode reveals the underlying transmission patterns of COVID-19 at the fine-scale in Hong Kong, China and the TransCode of Hong Kong, China can be adapted to characterize transmission patterns and predict transmission risks in other densely populated metropolises with limited data availability, and thus facilitating the accurate implementation of COVID-19 intervention strategies by uncovering the fine-scale transmission patterns in specific regions. In the future, we plan to extend our work from two perspectives. First, we aim to reach a finer scale for the characterization, e.g., blocks or buildings, for the purpose of more precise and effective control. Furthermore, we will take underdeveloped regions/countries into consideration, where the data scarcity problem presents more challenges.

## Supplementary Information

**Additional file 1.** Supplementary Material.

## Data Availability

All codes and data will be available from the authors upon reasonable request.
