# Supplementary material for: TransCode: Uncovering COVID-19 transmission patterns via deep learning
Source: Infect Dis Poverty. 2023 Feb 28;12:14. doi: 10.1186/s40249-023-01052-9 (PMC9971690; doi:10.1186/s40249-023-01052-9)
Supplement: Supplementary file 1 — Additional file 1. Supplementary Material. [file 40249_2023_1052_MOESM1_ESM.pdf]

Supplementary Material of "TransCode: Uncovering COVID-19 Transmission Patterns via Deep Learning"

Jinfu Ren[1], Mutong Liu[1], Yang Liu[1], Jiming Liu[1*]

[1]Department of Computer Science, Hong Kong Baptist University, Hong Kong SAR, China

*Correspondence to: J. Liu (jiming@comp.hkbu.edu.hk)

Table of Contents

Theoretical analysis of deep transfer learning model 2
1.1 The adaptability of TransCode 2
1.2 Mild conditions for performance guarantee on the target domain 2
Data resolution 4
Chord diagrams of the discovered TransCode in Hong Kong 4
3.1 Period 1 4
3.2 Period 3 5
Results of case number prediction 5
4.1 One-week ahead prediction results 5
4.1.1 Numerical results in terms of Mean Absolute Error (MAE) 5
4.1.2 Numerical results in terms of Relative Mean Absolute Error (RMAE) 7
4.2 Two-weeks-ahead prediction results 8
4.2.1 Numerical results in terms of Mean Absolute Error (MAE) 8
4.2.2 Numerical results in terms of Relative Mean Absolute Error (RMAE) 10
Inferred TransCodes of all metropolises 12
5.1 New York City 12
5.2 San Francisco 13
5.3 Toronto 14
5.4 London 15
5.5 Berlin 16
5.6 Tokyo 17
5.7 Hong Kong 18
Supplementary references 18

1 Theoretical analysis of deep transfer learning model

In this section, we present the theoretical analysis of the proposed deep transfer learning model to discuss the adaptability of TransCode. Note that the source domain and the target domain used in the following analysis refer to the source city and the target city, respectively, and the alphabets used here are independent from the main paper.

1.1 The adaptability of TransCode

To infer the TransCode in data-limited regions (the target domain), we adapt the TransCode obtained from a data-rich region (Hong Kong, the source domain) via the proposed deep transfer learning model. Intuitively, if the performance (i.e., the prediction accuracy) of the learning model on the source domain is guaranteed and the discrepancy between the data distribution of the source domain and that of the target domain is small enough, we can expect that the model performance on the target domain is also guaranteed. Mathematically, we have the following theorem to guarantee the adaptability of the TransCode, i.e., the performance of the proposed deep transfer learning model with the adapted TransCode on the target domain (city).

Theorem 1. *Assume that the prediction error of the learning model L over the training data set* $\mathcal{C}$ *is bounded by* $M = sup_{\mathcal{C}} sup_{x\in\mathcal{C},h\in\mathcal{H}} L\big(h(x), f_{\mathcal{C}}(x)\big)$*, where* $\mathcal{H}$ *is the hypothesis class and* $f_{\mathcal{C}}(\cdot)$ *is the ideal hypothesis, then the performance of model L with the adapted TransCode on the target domain is bounded by the following inequality:*

$$\epsilon_t(h, f_t) \leq \epsilon_s(h, f_s) + \eta_{\mathcal{H}}(f_s, f_t) + d_{\mathcal{A}}(\widehat{P_X}, \widehat{Q_X}), \quad (1)$$

where $\epsilon_t(h, f_t) = \mathbb{E}_{x\sim P}\big[L\big(h(x), f_t(x)\big)\big]$ *and* $\epsilon_s(h, f_s) = \mathbb{E}_{x\sim Q}\big[L\big(h(x), f_s(x)\big)\big]$ *are the average prediction error of model L on the target domain and that on the source domain, respectively,* $\eta_{\mathcal{H}}(f_s, f_t) = min_{h_0\in\mathcal{H}}[\epsilon_t(h_0, f_t) + \epsilon_s(h_0, f_s)]$ *is the sum of the prediction error produced by the ideal hypothesis over the training data set,* P_X *and* Q_X *are true data distributions of the target domain and the source domain, respectively,* $\widehat{P_X}$ *and* $\widehat{Q_X}$ *are the distributions of the observations that drawn from* P_X *and* Q_X*, respectively, and* $d_{\mathcal{A}}(\widehat{P_X}, \widehat{Q_X}) = 2sup_{A\in\mathcal{A}}\big|\widehat{P_X}(A) - \widehat{Q_X}(A)\big|$ *is the* $d_{\mathcal{A}}$ *distance between* $\widehat{P_X}$ *and* $\widehat{Q_X}$*.*

As mentioned above, the left-hand side of the inequality, which is used to quantify the performance of the proposed model on the target domain, is bounded by the sum of three components on the right-hand side: the model performance on the source domain (the first term), the performance of ideal hypotheses on both domains (the second term), and the discrepancy between the observation distribution of source domain and that of target domain (the third term). In the following, we first prove the Theorem 1. Then we discuss the condition under which the second and third terms on the right-hand side in Eq. (1) could achieve a small value, resulting in a well bounded error of the model on target domain.

Proof of Theorem 1:

$$\begin{aligned}
\epsilon_t(h, f_t) &\leq \epsilon_s(h, f_s) + |\epsilon_t(h, f_t) - \epsilon_s(h, f_s)| \\
&\leq \epsilon_s(h, f_s) + |\epsilon_t(h, h_0) - \epsilon_t(h, f_t)| + |\epsilon_s(h, h_0) - \epsilon_s(h, f_s)| + |\epsilon_t(h, h_0) - \epsilon_s(h, h_0)| \\
&\leq \epsilon_s(h, f_s) + max_{h,h_0\in\mathcal{H}}\big|\mathbb{E}_{x\sim P}\big[L\big(h(x), h_0(x)\big)\big] - \mathbb{E}_{x\sim Q}\big[L\big(h(x), h_0(x)\big)\big]\big| + \\
&\quad \big[\big|L\big(h(x), h_0(x)\big) - L\big(h(x), f_t(x)\big)\big|\big] + \mathbb{E}_{x\sim Q}\big[\big|L\big(h(x), h_0(x)\big) - L\big(h(x), f_s(x)\big)\big|\big] \\
&\leq \epsilon_s(h, f_s) + max_{h,h_0\in\mathcal{H}}\big|\mathbb{E}_{x\sim P}\big[L\big(h(x), h_0(x)\big)\big] - \mathbb{E}_{x\sim Q}\big[L\big(h(x), h_0(x)\big)\big]\big| + \\
&\quad \mathbb{E}_{x\sim P}\big[\big|L\big(h_0(x), f_t(x)\big)\big|\big] + \mathbb{E}_{x\sim Q}\big[\big|L\big(h_0(x), f_s(x)\big)\big|\big] \\
&= \epsilon_s(h, f_s) + max_{h,h_0\in\mathcal{H}}\big|\mathbb{E}_{x\sim P}\big[L\big(h(x), h_0(x)\big)\big] - \mathbb{E}_{x\sim Q}\big[L\big(h(x), h_0(x)\big)\big]\big| + \\
&\quad min_{h_0\in\mathcal{H}}[\epsilon_t(h_0, f_t) + \epsilon_s(h_0, f_s)] \\
&\leq \epsilon_s(h, f_s) + d_{\mathcal{A}}(\widehat{P_X}, \widehat{Q_X}) + min_{h_0\in\mathcal{H}}[\epsilon_t(h_0, f_t) + \epsilon_s(h_0, f_s)],
\end{aligned} \quad (2)$$

where h_0 is any hypothesis that satisfies $h_0 \in \mathcal{H}$, and $h(x)$ is the prediction result of the input x generated by h. Moreover, $\mathbb{E}_{x\sim Q}\big[L\big(h(x), h_0(x)\big)\big]$ and $\mathbb{E}_{x\sim P}\big[L\big(h(x), h_0(x)\big)\big]$ denote the expectation of difference between h_0 and h over the training data on source domain and that on target domain, respectively.

■

1.2 Mild conditions for performance guarantee on the target domain

To guarantee that the model performance on the target domain is close to that on the source domain, the last two terms on the right-hand side of Eq. (1) should be small. To satisfy this requirement, the following two mild conditions are expected to be held (according to the impossibility theorem[1]).

(Condition 1) The similarity between data distributions: The following $d_{\mathcal{A}}$ distance between $\widehat{P_X}$ and $\widehat{Q_X}$ should be as small as possible:

$$d_{\mathcal{A}}(\widehat{P_X}, \widehat{Q_X}) = 2sup_{A\in\mathcal{A}}\left|\widehat{P_X}(A) - \widehat{Q_X}(A)\right|, \tag{3}$$

where $\mathcal{A}$ is the collection of subsets of X such that each subset is measurable with respect to $\widehat{P_X}$ and $\widehat{Q_X}$.

(Condition 2) Low joint error: There should exist a low-error hypothesis for both domains. The error is defined as follows:

$$\begin{aligned} \lambda_{\mathcal{H}} &= min_{h\in\mathcal{H}} R_T(h) + R_S(h) \\ &= min_{h\in\mathcal{H}} \mathbb{E}_{x\sim T}\left[L\big(h(x), f_T(x)\big)\right] + \mathbb{E}_{x\sim S}\left[L\big(h(x), f_S(x)\big)\right] \\ &= min_{h\in\mathcal{H}}[\epsilon_t(h, f_t) + \epsilon_s(h, f_s)], \end{aligned} \tag{4}$$

where $R_T(h) = \mathbb{E}_{(x,y)\sim T}\left[L\big(h(x), f_T(x)\big)\right]$ measures the error of a hypothesis h on domain T.

For Condition 1, since the $d_{\mathcal{A}}(\widehat{P_X}, \widehat{Q_X})$ is difficult to calculate, we instead measure its upper bound. According to Kifer et al[2] and Cai et al[4], we have $d_{\mathcal{A}}(\widehat{P_X}, \widehat{Q_X}) \le d_{L_1}(\widehat{P_X}, \widehat{Q_X}) \le \sqrt{0.5JS(\widehat{P_X}, \widehat{Q_X})}$, where $d_{L_1}(\cdot,\cdot)$ is the L_1 distance and $JS(\cdot,\cdot)$ is the Jensen-Shannon (JS) divergence[5]. Therefore, if the JS divergence is small, then the $d_{\mathcal{A}}$ distance will also be small. In our case, Hong Kong is the data-rich source domain, New York City, San Francisco, Toronto, London, Berlin and Tokyo are data-limited target domain, and $\widehat{Q_X}$ and $\widehat{P_X}$ are the distribution of case number in the source domain and that in the target domain, respectively. We then calculate the JS divergence for each source-target pair and obtain the following results:
$JS\big(\widehat{P_X}, \widehat{Q_X}\big) = 0.35$ (with New York City being the target city),
$JS\big(\widehat{P_X}, \widehat{Q_X}\big) = 0.10$ (with San Francisco being the target city),
$JS\big(\widehat{P_X}, \widehat{Q_X}\big) = 0.17$ (with Toronto being the target city),
$JS\big(\widehat{P_X}, \widehat{Q_X}\big) = 0.16$ (with London being the target city),
$JS\big(\widehat{P_X}, \widehat{Q_X}\big) = 0.14$ (with Berlin being the target city), and
$JS\big(\widehat{P_X}, \widehat{Q_X}\big) = 0.15$ (with Tokyo being the target city).

For comparison, we also calculate the JS divergence between Hong Kong and another two cities, Santa Fe and Cordoba in Argentina, which are not that densely populated compared with the aforementioned six metropolises. We have:
$JS\big(\widehat{P_X}, \widehat{Q_X}\big) = 0.59$ (with Santa Fe being the target city) and
$JS\big(\widehat{P_X}, \widehat{Q_X}\big) = 0.57$ (with Cordoba being the target city),
which are significantly larger than the JS divergence on New York City, San Francisco, Toronto, London, Berlin and Tokyo. As discussed above, small JS divergence between source and target domains is preferred to achieve good adaptation performance. This is also consistent with our experimental results: by adapting the TransCode from a source domain with similar data distribution (i.e., Hong Kong), the proposed deep transferred learning model performs the best among all five methods on the target domain (six densely populated metropolises).

For Condition 2, according to the definition of $\eta_{\mathcal{H}}$ given in Theorem 1, $\lambda_{\mathcal{H}}$ in Eq. (4) is equivalent to $\eta_{\mathcal{H}}$. As a result, the second term on the right-hand side in Eq. (1) is guaranteed to be small if the Condition 2 is satisfied. In fact, $\lambda_{\mathcal{H}}$ could be considered as a measurement of the agreement between the label distribution on target domain and that on source domain[2]. In our case, the label distribution on target domain and that on source domain are directly sampled from $\widehat{P_X}$ and $\widehat{Q_X}$, respectively, whose similarity can be measured using a similar manner as described in Eq. (3).

2 Data resolution

The number of the fine-scale districts/boroughs in seven metropolises, namely New York City, San Francisco, Toronto, London, Berlin, Tokyo and Hong Kong, is given in the Supplementary Table 1.

Supplementary Table 1 ***The number of the fine-scale districts/boroughs of New York City, San Francisco, Toronto, London, Berlin, Tokyo and Hong Kong.***

Order	City Name	Spatial resolution
1	New York City	5 boroughs
2	San Francisco	27 census zip code tabulation areas
3	Toronto	25 electoral wards
4	London	32 boroughs
5	Berlin	12 districts
6	Tokyo	62 districts
7	Hong Kong	18 districts, 452 constituencies

3 Chord diagrams of the discovered TransCode in Hong Kong

3.1 Period 1

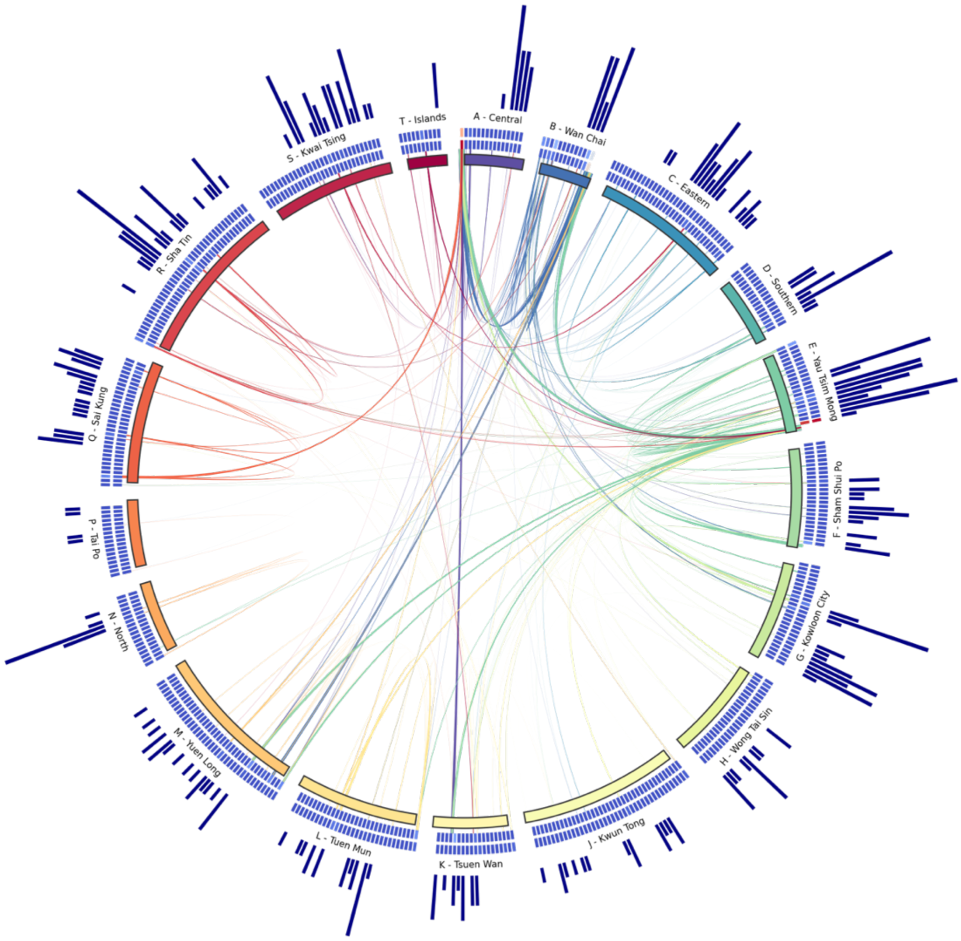

Supplementary Figure 1 ***Chord diagram of the Hong Kong TransCode (period 1) at the constituency level.*** *The outermost bar charts show the cumulative case numbers in the 452 constituencies. Next to the bar charts, the two-layer heatmaps illustrate the exported (outside layer) and imported (inside layer) disease transmission intensities of each constituency, respectively. Red indicates high transmission intensity, and blue indicates low transmission intensity. Next to the heatmaps, the inner circle of the chord diagram is divided into 18 pieces, each a different color, representing the districts. The innermost connections show the disease transmission between different constituencies. The color of the edge shows the district to which the constituency belongs, and the width of the edge denotes the transmission intensity.*

3.2 Period 3

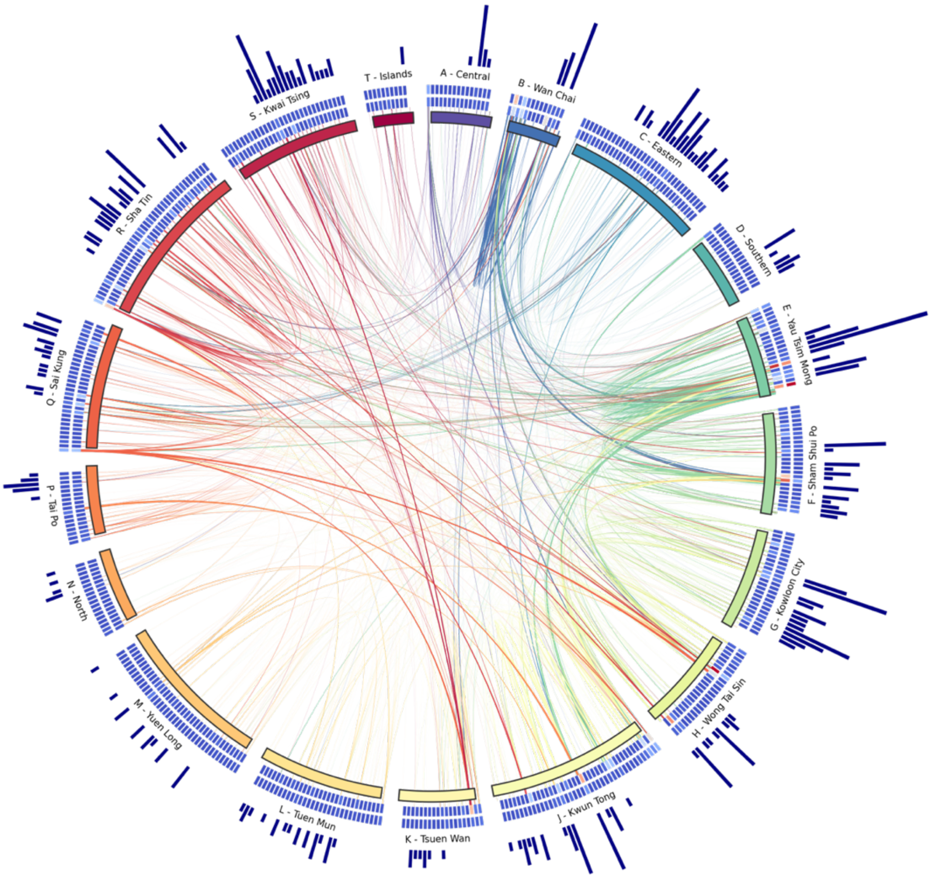

Supplementary Figure 2 ***Chord diagram of the Hong Kong TransCode (period 3) at the constituency level.*** *The outermost bar charts show the cumulative case numbers in the 452 constituencies. Next to the bar charts, the two-layer heatmaps illustrate the exported (outside layer) and imported (inside layer) disease transmission intensities of each constituency, respectively. Red indicates high transmission intensity, and blue indicates low transmission intensity. Next to the heatmaps, the inner circle of the chord diagram is divided into 18 pieces, each a different color, representing the districts. The innermost connections show the disease transmission between different constituencies. The color of the edge shows the district to which the constituency belongs, and the width of the edge denotes the transmission intensity.*

4 Results of case number prediction

4.1 One-week ahead prediction results

4.1.1 Numerical results in terms of Mean Absolute Error (MAE)

Supplementary Table 2 ***MAE of the one-week-ahead district-level case number prediction of SEIR, MRPMC, LSTM-T, Informer, and our method of different periods in New York City.*** *The best performance is highlighted in* ***bold.***

NYC					
	SEIR	MRPMC	LSTM-T	Informer	Our Method
Period 1 02/29/20~09/02/20	50	187.84	122.07	21.27	**17.84**
Period 2 09/02/20~04/10/21	516.92	232.07	227.73	282.41	**198.42**
Period 3 04/10/21~09/01/21	295.31	91.30	133.78	122.91	**67.69**

Supplementary Table 3 ***MAE of the one-week-ahead district-level case number prediction of SEIR, MRPMC, LSTM-T, Informer, and our method of different periods in San Francisco.*** *The best performance is highlighted in* **bold.**

San Francisco					
	SEIR	MRPMC	LSTM-T	Informer	Our Method
Period 1 03/03/20~08/12/20	3.34	8.23	12.05	5.49	**2.68**
Period 2 08/12/20~01/05/21	9.87	16.74	24.27	13.07	**4.89**
Period 3 01/05/21~05/10/21	0.70	12.73	23.98	2.62	**0.65**
Period 4 05/10/21~08/27/21	5.02	10.60	19.38	5.33	**2.76**

Supplementary Table 4 ***MAE of the one-week-ahead district-level case number prediction of SEIR, MRPMC, LSTM-T, Informer, and our method of different periods in Toronto.*** *The best performance is highlighted in* **bold.**

Toronto					
	SEIR	MRPMC	LSTM-T	Informer	Our Method
Period 1 03/01/20~08/30/20	1.16	2.48	1.61	1.18	**0.76**
Period 2 08/30/20~02/13/21	16.50	7.84	12.73	8.68	**6.22**
Period 3 02/13/21~05/24/21	21.49	9.57	13.36	12.05	**8.03**
Period 4 05/24/21~09/02/21	5.82	6.50	2.78	3.00	**2.46**

Supplementary Table 5 ***MAE of the one-week-ahead district-level case number prediction of SEIR, MRPMC, LSTM-T, Informer, and our method of different periods in London.*** *The best performance is highlighted in* **bold.**

London					
	SEIR	MRPMC	LSTM-T	Informer	Our Method
Period 1 02/11/20~06/21/20	1.47	5.13	4.50	5.23	**1.18**
Period 2 06/21/20~11/28/20	67.95	52.46	26.17	49.17	**12.42**
Period 3 11/28/20~05/06/21	7.76	51.91	17.97	8.93	**3.99**
Period 4 05/06/21~09/02/21	96.45	42.66	25.58	28.29	**24.92**

Supplementary Table 6 ***MAE of the one-week-ahead district-level case number prediction of SEIR, MRPMC, LSTM-T, Informer, and our method of different periods in Berlin.*** *The best performance is highlighted in* **bold.**

Berlin					
	SEIR	MRPMC	LSTM-T	Informer	Our Method
Period 1 03/16/20~09/14/20	5.16	2.71	3.63	4.29	**2.14**
Period 2 09/14/20~03/09/21	29.07	6.26	11.07	13.60	**5.32**
Period 3 03/09/21~09/02/21	32.67	10.70	15.63	13.89	**7.07**

Supplementary Table 7 ***MAE of the one-week-ahead district-level case number prediction of SEIR, MRPMC, LSTM-T, Informer, and our method of different periods in Tokyo.*** *The best performance is highlighted in* ***bold.***

Tokyo					
	SEIR	MRPMC	LSTM-T	Informer	Our Method
Period 1 03/31/20~09/17/20	2.28	2.76	3.02	1.73	**1.07**
Period 2 09/17/20~01/15/21	25.61	23.51	24.40	20.13	**14.43**
Period 3 01/15/21~06/05/21	6.79	7.44	7.78	6.88	**3.53**
Period 4 06/05/21~09/02/21	66.38	59.05	55.96	47.51	**12.93**

4.1.2 Numerical results in terms of Relative Mean Absolute Error (RMAE)

Supplementary Table 8 ***RMAE of the one-week-ahead district-level case number prediction of SEIR, MRPMC, LSTM-T, Informer, and our method of different periods in New York City.*** *The best performance is highlighted in* ***bold.***

NYC					
	SEIR	MRPMC	LSTM-T	Informer	Our Method
Period 1 02/29/20~09/02/20	1	3.75	2.44	0.42	**0.35**
Period 2 09/02/20~04/10/21	0.99	0.44	0.44	0.54	**0.38**
Period 3 04/10/21~09/01/21	1	0.30	0.45	0.41	**0.22**

Supplementary Table 9 ***RMAE of the one-week-ahead district-level case number prediction of SEIR, MRPMC, LSTM-T, Informer, and our method of different periods in San Francisco.*** *The best performance is highlighted in* ***bold.***

San Francisco					
	SEIR	MRPMC	LSTM-T	Informer	Our Method
Period 1 03/03/20~08/12/20	1	2.47	3.60	1.64	**0.80**
Period 2 08/12/20~01/05/21	1	1.69	2.45	1.32	**0.49**
Period 3 01/05/21~05/10/21	1	18.18	34.25	3.74	**0.92**
Period 4 05/10/21~08/27/21	0.99	2.10	3.86	1.05	**0.54**

Supplementary Table 10 ***RMAE of the one-week-ahead district-level case number prediction of SEIR, MRPMC, LSTM-T, Informer, and our method of different periods in Toronto.*** *The best performance is highlighted in* ***bold.***

Toronto					
	SEIR	MRPMC	LSTM-T	Informer	Our Method
Period 1 03/01/20~08/30/20	0.98	2.10	1.36	1	**0.64**
Period 2 08/30/20~02/13/21	1	0.47	0.77	0.52	**0.37**
Period 3 02/13/21~05/24/21	0.99	0.44	0.62	0.55	**0.37**
Period 4 05/24/21~09/02/21	0.99	1.11	0.48	0.51	**0.42**

Supplementary Table 11 ***RMAE of the one-week-ahead district-level case number prediction of SEIR, MRPMC, LSTM-T, Informer, and our method of different periods in London.*** *The best performance is highlighted in* ***bold.***

London					
	SEIR	MRPMC	LSTM-T	Informer	Our Method
Period 1 02/11/20~06/21/20	0.98	3.44	3.04	3.51	**0.79**
Period 2 06/21/20~11/28/20	0.99	0.77	0.38	0.72	**0.18**
Period 3 11/28/20~05/06/21	0.99	6.68	2.31	1.14	**0.51**
Period 4 05/06/21~09/02/21	0.99	0.44	0.26	0.29	**0.25**

Supplementary Table 12 ***RMAE of the one-week-ahead district-level case number prediction of SEIR, MRPMC, LSTM-T, Informer, and our method of different periods in Berlin.*** *The best performance is highlighted in* ***bold.***

Berlin					
	SEIR	MRPMC	LSTM-T	Informer	Our Method
Period 1 03/16/20~09/14/20	0.8	0.42	0.56	0.66	**0.33**
Period 2 09/14/20~03/09/21	0.95	0.20	0.36	0.44	**0.17**
Period 3 03/09/21~09/02/21	0.96	0.31	0.46	0.40	**0.20**

Supplementary Table 13 ***RMAE of the one-week-ahead district-level case number prediction of SEIR, MRPMC, LSTM-T, Informer, and our method of different periods in Tokyo.*** *The best performance is highlighted in* ***bold.***

Tokyo					
	SEIR	MRMPC	LSTM-T	Informer	Our Method
Period 1 03/31/20~09/17/20	0.78	0.94	1.03	0.59	**0.36**
Period 2 09/17/20~01/15/21	0.95	0.87	0.90	0.74	**0.53**
Period 3 01/15/21~06/05/21	0.72	0.79	0.83	0.73	**0.37**
Period 4 06/05/21~09/02/21	0.95	0.84	0.80	0.68	**0.18**

4.2 Two-weeks-ahead prediction results

4.2.1 Numerical results in terms of Mean Absolute Error (MAE)

Supplementary Table 14 ***MAE of the two-weeks-ahead district-level case number prediction of SEIR, MRPMC, LSTM-T, Informer, and our method of different periods in New York City.*** *The best performance is highlighted in* ***bold.***

NYC					
	SEIR	MRPMC	LSTM-T	Informer	Our Method
Period 1 02/29/20~09/02/20	48.37	180.37	133.02	20.17	**17.53**
Period 2 09/02/20~04/10/21	553.4	281.73	380.04	341.13	**209.90**
Period 3 04/10/21~09/01/21	300.03	95.88	138.52	100.83	**43.06**

Supplementary Table 15 ***MAE of the two-weeks-ahead district-level case number prediction of SEIR, MRPMC, LSTM-T, Informer, and our method of different periods in San Francisco.*** *The best performance is highlighted in* ***bold.***

San Francisco					
	SEIR	MRPMC	LSTM-T	Informer	Our Method
Period 1 03/03/20~08/12/20	3.41	9.18	11.90	5.59	**2.91**
Period 2 08/12/20~01/05/21	9.03	17.71	14.34	12.13	**6.96**
Period 3 01/05/21~05/10/21	0.83	16.65	24.15	2.67	**0.70**
Period 4 05/10/21~08/27/21	5.55	8.89	18.49	5.86	**3.53**

Supplementary Table 16 ***MAE of the two-weeks-ahead district-level case number prediction of SEIR, MRPMC, LSTM-T, Informer, and our method of different periods in Toronto.*** *The best performance is highlighted in* ***bold.***

Toronto					
	SEIR	MRPMC	LSTM-T	Informer	Our Method
Period 1 03/01/20~08/30/20	1.01	2.57	0.79	1.25	**0.69**
Period 2 08/30/20~02/13/21	19.48	10.54	7.89	10.90	**6.18**
Period 3 02/13/21~05/24/21	25.28	12.12	17.03	13.44	**6.43**
Period 4 05/24/21~09/02/21	5.47	7.23	2.87	4.43	**2.36**

Supplementary Table 17 ***MAE of the two-weeks-ahead district-level case number prediction of SEIR, MRPMC, LSTM-T, Informer, and our method of different periods in London.*** *The best performance is highlighted in* ***bold.***

London					
	SEIR	MRPMC	LSTM-T	Informer	Our Method
Period 1 02/11/20~06/21/20	1.56	6.05	4.50	4.87	**0.81**
Period 2 06/21/20~11/28/20	73.37	61.24	62.33	55.56	**16.90**
Period 3 11/28/20~05/06/21	8.62	50.40	44.53	12.82	**5.43**
Period 4 05/06/21~09/02/21	100.51	48.28	26.53	40.70	**18.05**

Supplementary Table 18 ***MAE of the two-weeks-ahead district-level case number prediction of SEIR, MRPMC, LSTM-T, Informer, and our method of different periods in Berlin.*** *The best performance is highlighted in* ***bold.***

Berlin					
	SEIR	MRPMC	LSTM-T	Informer	Our Method
Period 1 03/16/20~09/14/20	4.87	2.71	3.40	7.01	**2.07**
Period 2 09/14/20~03/09/21	27.43	6.08	9.13	21.28	**5.63**
Period 3 03/09/21~09/02/21	32.04	9.69	14.99	19.58	**4.58**

Supplementary Table 19 ***MAE of the two-weeks-ahead district-level case number prediction of SEIR, MRPMC, LSTM-T, Informer, and our method of different periods in Tokyo.*** *The best performance is highlighted in* ***bold.***

Tokyo					
	SEIR	MRPMC	LSTM-T	Informer	Our Method
Period 1 03/31/20~09/17/20	2.35	2.77	2.66	1.70	**1.11**
Period 2 09/17/20~01/15/21	19.81	18.06	15.52	15.00	**13.06**
Period 3 01/15/21~06/05/21	7.63	8.09	8.16	5.33	**2.58**
Period 4 06/05/21~09/02/21	73.07	66.10	60.78	56.21	**19.85**

4.2.2 Numerical results in terms of Relative Mean Absolute Error (RMAE)

Supplementary Table 20 ***RMAE of the two-weeks-ahead district-level case number prediction of SEIR, MRPMC, LSTM-T, Informer, and our method of different periods in New York City.*** *The best performance is highlighted in* ***bold.***

NYC					
	SEIR	MRPMC	LSTM-T	Informer	Our Method
Period 1 02/29/20~09/02/20	1	3.72	2.75	0.41	**0.36**
Period 2 09/02/20~04/10/21	1	0.50	0.68	0.61	**0.37**
Period 3 04/10/21~09/01/21	1	0.31	0.46	0.33	**0.14**

Supplementary Table 21 ***RMAE of the two-weeks-ahead district-level case number prediction of SEIR, MRPMC, LSTM-T, Informer, and our method of different periods in San Francisco.*** *The best performance is highlighted in* ***bold.***

San Francisco					
	SEIR	MRPMC	LSTM-T	Informer	Our Method
Period 1 03/03/20~08/12/20	0.98	2.64	3.48	1.61	**0.83**
Period 2 08/12/20~01/05/21	1	1.96	1.58	1.34	**0.77**
Period 3 01/05/21~05/10/21	1	20.06	29.09	3.21	**0.84**
Period 4 05/10/21~08/27/21	1	1.60	3.33	1.05	**0.63**

Supplementary Table 22 ***RMAE of the two-weeks-ahead district-level case number prediction of SEIR, MRPMC, LSTM-T, Informer, and our method of different periods in Toronto.*** *The best performance is highlighted in* ***bold.***

Toronto					
	SEIR	MRPMC	LSTM-T	Informer	Our Method
Period 1 03/01/20~08/30/20	0.99	2.51	0.77	1.22	**0.67**
Period 2 08/30/20~02/13/21	0.99	0.54	0.40	0.55	**0.31**
Period 3 02/13/21~05/24/21	0.99	0.47	0.67	0.53	**0.25**
Period 4 05/24/21~09/02/21	0.99	1.31	0.52	0.80	**0.42**

Supplementary Table 23 ***RMAE of the two-weeks-ahead district-level case number prediction of SEIR, MRPMC, LSTM-T, Informer, and our method of different periods in London.*** *The best performance is highlighted in* ***bold.***

London					
	SEIR	MRPMC	LSTM-T	Informer	Our Method
Period 1<br>02/11/20~06/21/20	0.98	3.82	2.87	3.08	**0.51**
Period 2<br>06/21/20~11/28/20	0.99	0.83	0.84	0.75	**0.23**
Period 3<br>11/28/20~05/06/21	0.99	5.82	5.15	1.48	**0.62**
Period 4<br>05/06/21~09/02/21	0.99	0.48	0.26	0.40	**0.18**

Supplementary Table 24 ***RMAE of the two-weeks-ahead district-level case number prediction of SEIR, MRPMC, LSTM-T, Informer, and our method of different periods in Berlin.*** *The best performance is highlighted in* ***bold.***

Berlin					
	SEIR	MRPMC	LSTM-T	Informer	Our Method
Period 1<br>03/16/20~09/14/20	0.79	0.43	0.55	1.13	**0.33**
Period 2<br>09/14/20~03/09/21	0.95	0.21	0.31	0.74	**0.19**
Period 3<br>03/09/21~09/02/21	0.96	0.29	0.44	0.58	**0.13**

Supplementary Table 25 ***RMAE of the two-weeks-ahead district-level case number prediction of SEIR, MRPMC, LSTM-T, Informer, and our method of different periods in Tokyo.*** *The best performance is highlighted in* ***bold.***

Tokyo					
	SEIR	MRPMC	LSTM-T	Informer	Our Method
Period 1<br>03/31/20~09/17/20	0.79	0.93	0.89	0.57	**0.37**
Period 2<br>09/17/20~01/15/21	0.94	0.85	0.73	0.71	**0.62**
Period 3<br>01/15/21~06/05/21	0.75	0.80	0.80	0.52	**0.25**
Period 4<br>06/05/21~09/02/21	0.96	0.86	0.79	0.73	**0.26**

5 Inferred TransCodes of all metropolises

5.1 New York City

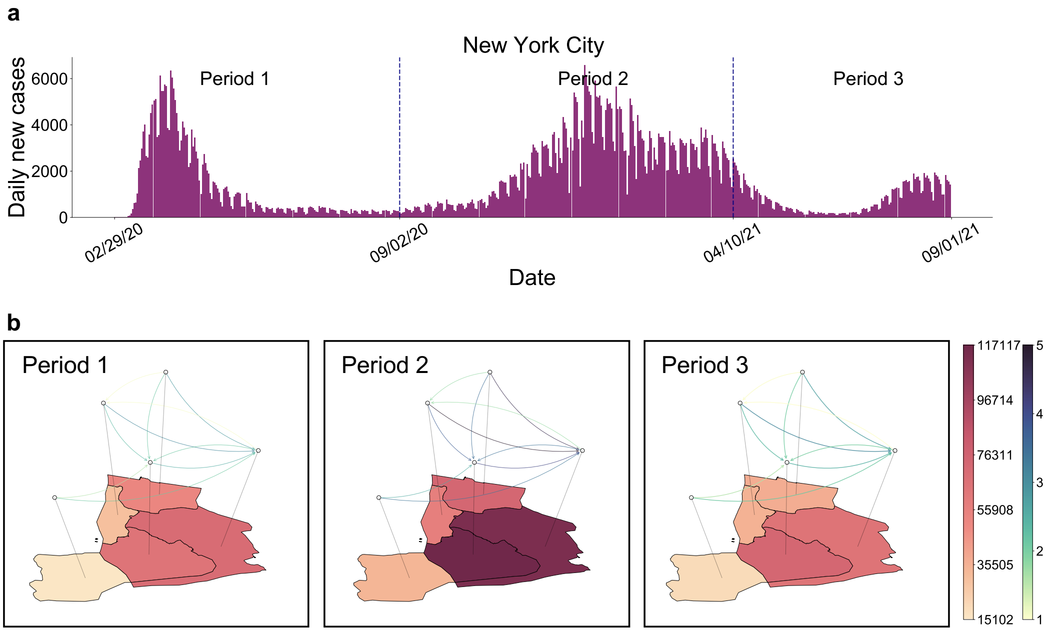

Supplementary Figure 3 ***Results of temporal clustering of the case dynamics and the inferred TransCode for each period in New York City.*** *(a) The sequence is partitioned into three periods, demonstrating different temporal patterns. (b) Visualization of the inferred TransCodes for three periods in New York City. In each subfigure, the map shows the number of cumulative confirmed cases in each district during the corresponding period (darker colors indicate higher case numbers); the transmission network represents the inferred TransCode. The network nodes correspond to the districts, and the directed network edges show transmission from one district to another (darker edge colors indicate higher transmission intensities).*

5.2 San Francisco

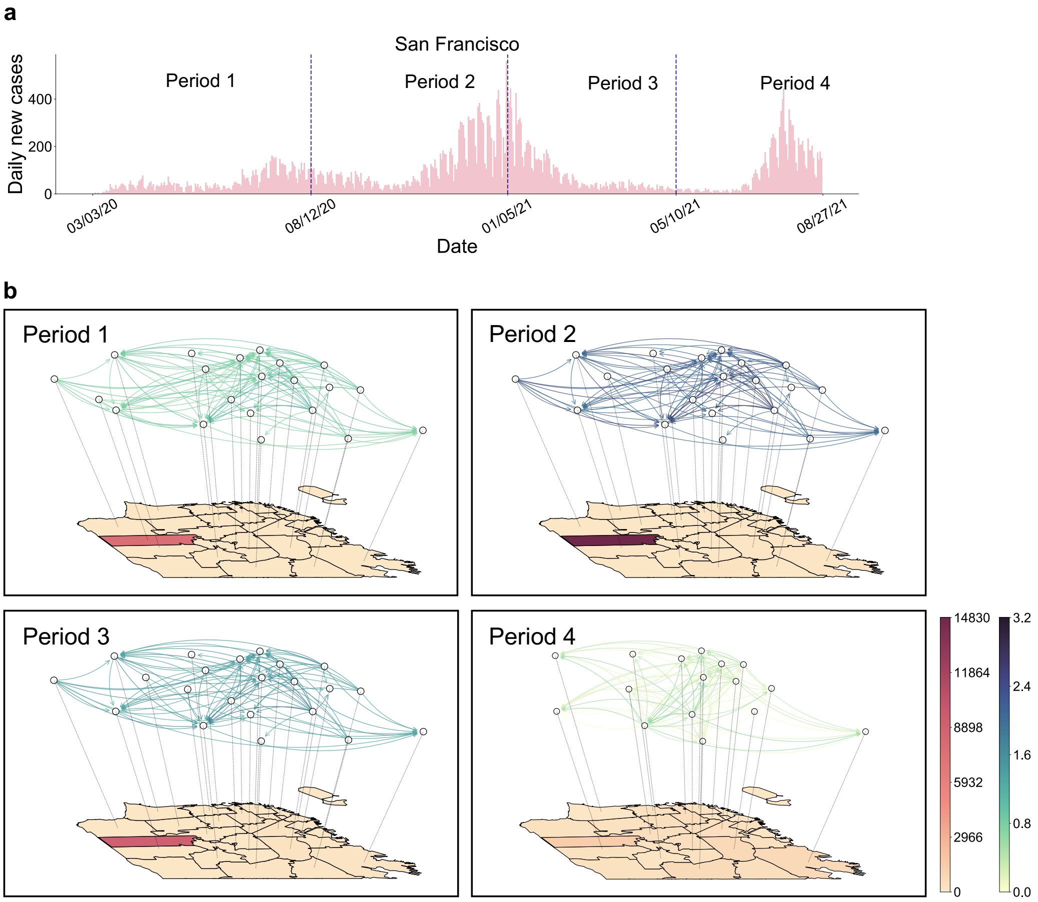

Supplementary Figure 4 ***Results of temporal clustering of the case dynamics and the inferred TransCode for each period in San Francisco.*** *(a) The sequence is partitioned into four periods, demonstrating different temporal patterns. (b) Visualization of the inferred TransCodes for four periods in San Francisco. In each subfigure, the map shows the number of cumulative confirmed cases in each district during the corresponding period (darker colors indicate higher case numbers); the transmission network represents the inferred TransCode. The network nodes correspond to the districts, and the directed network edges show transmission from one district to another (darker edge colors indicate higher transmission intensities).*

5.3 Toronto

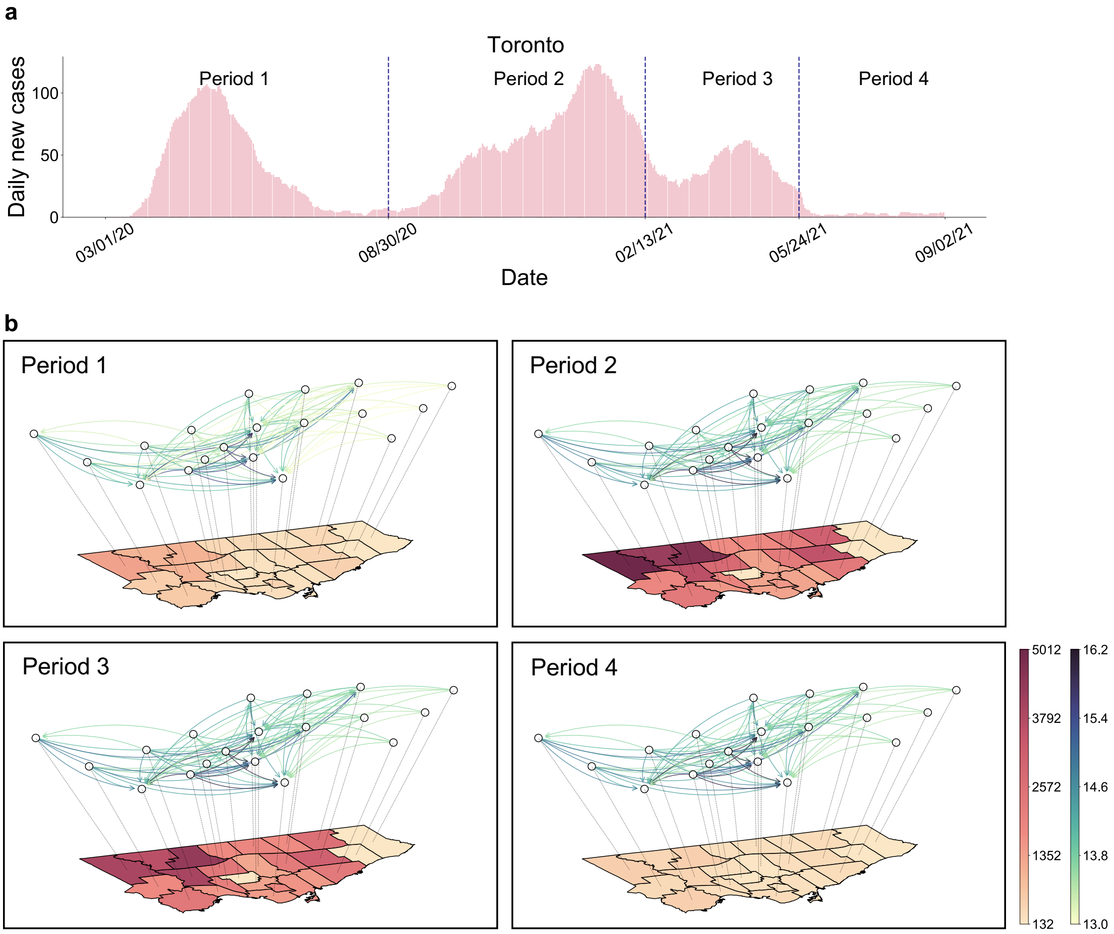

Supplementary Figure 5 ***Results of temporal clustering of the case dynamics and the inferred TransCode for each period in Toronto.*** *(a) The sequence is partitioned into four periods, demonstrating different temporal patterns. (b) Visualization of the inferred TransCodes for four periods in Toronto. In each subfigure, the map shows the number of cumulative confirmed cases in each district during the corresponding period (darker colors indicate higher case numbers); the transmission network represents the inferred TransCode. The network nodes correspond to the districts, and the directed network edges show transmission from one district to another (darker edge colors indicate higher transmission intensities).*

5.4 London

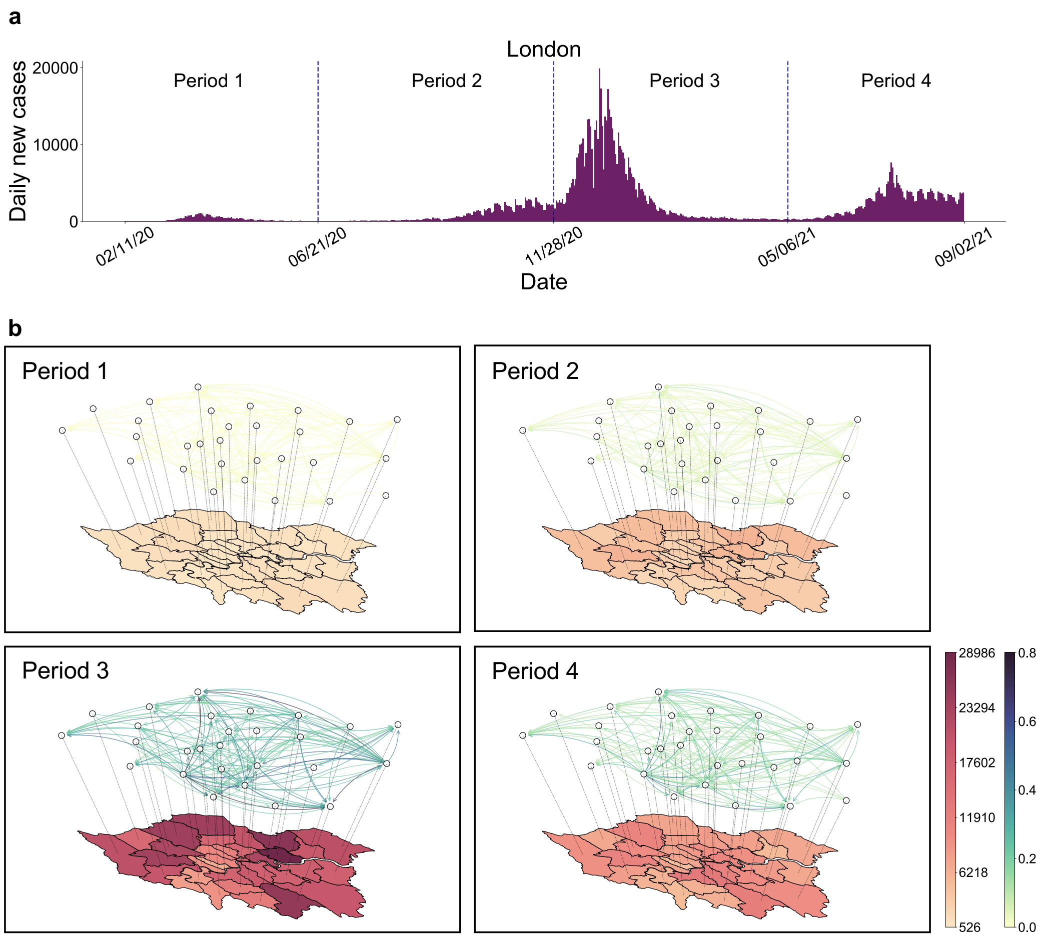

Supplementary Figure 6 ***Results of temporal clustering of the case dynamics and the inferred TransCode for each period in London.*** *(a) The sequence is partitioned into four periods, demonstrating different temporal patterns. (b) Visualization of the inferred TransCodes for four periods in London. In each subfigure, the map shows the number of cumulative confirmed cases in each district during the corresponding period (darker colors indicate higher case numbers); the transmission network represents the inferred TransCode. The network nodes correspond to the districts, and the directed network edges show transmission from one district to another (darker edge colors indicate higher transmission intensities).*

5.5 Berlin

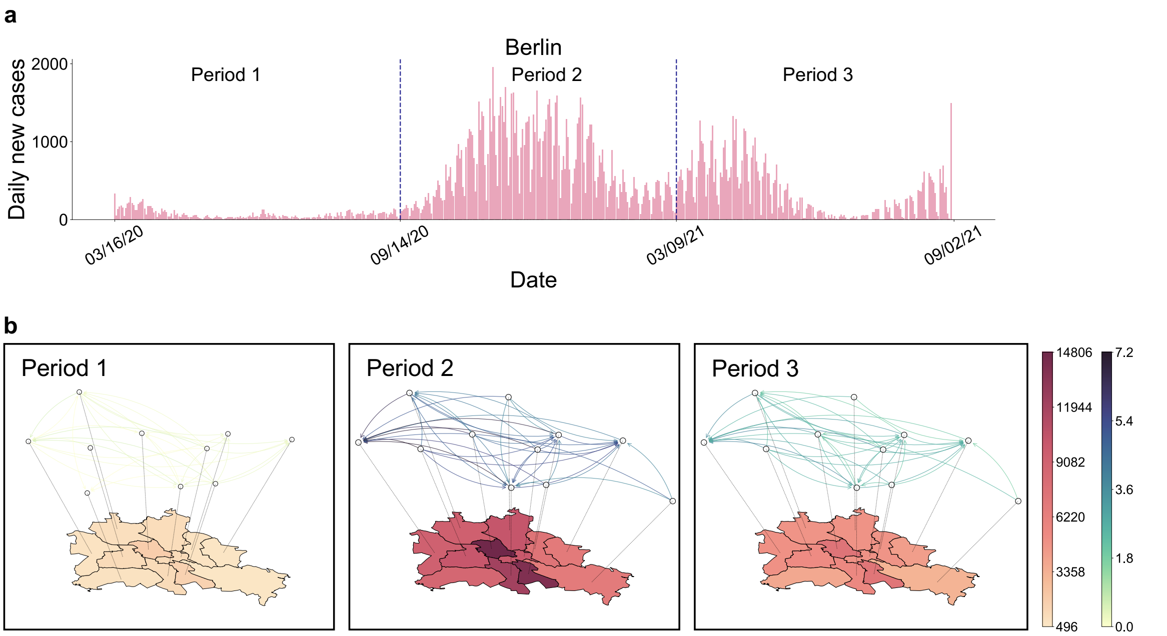

Supplementary Figure 7 ***Results of temporal clustering of the case dynamics and the inferred TransCode for each period in Berlin.*** *(a) The sequence is partitioned into three periods, demonstrating different temporal patterns. (b) Visualization of the inferred TransCodes for three periods in Berlin. In each subfigure, the map shows the number of cumulative confirmed cases in each district during the corresponding period (darker colors indicate higher case numbers); the transmission network represents the inferred TransCode. The network nodes correspond to the districts, and the directed network edges show transmission from one district to another (darker edge colors indicate higher transmission intensities).*

5.6 Tokyo

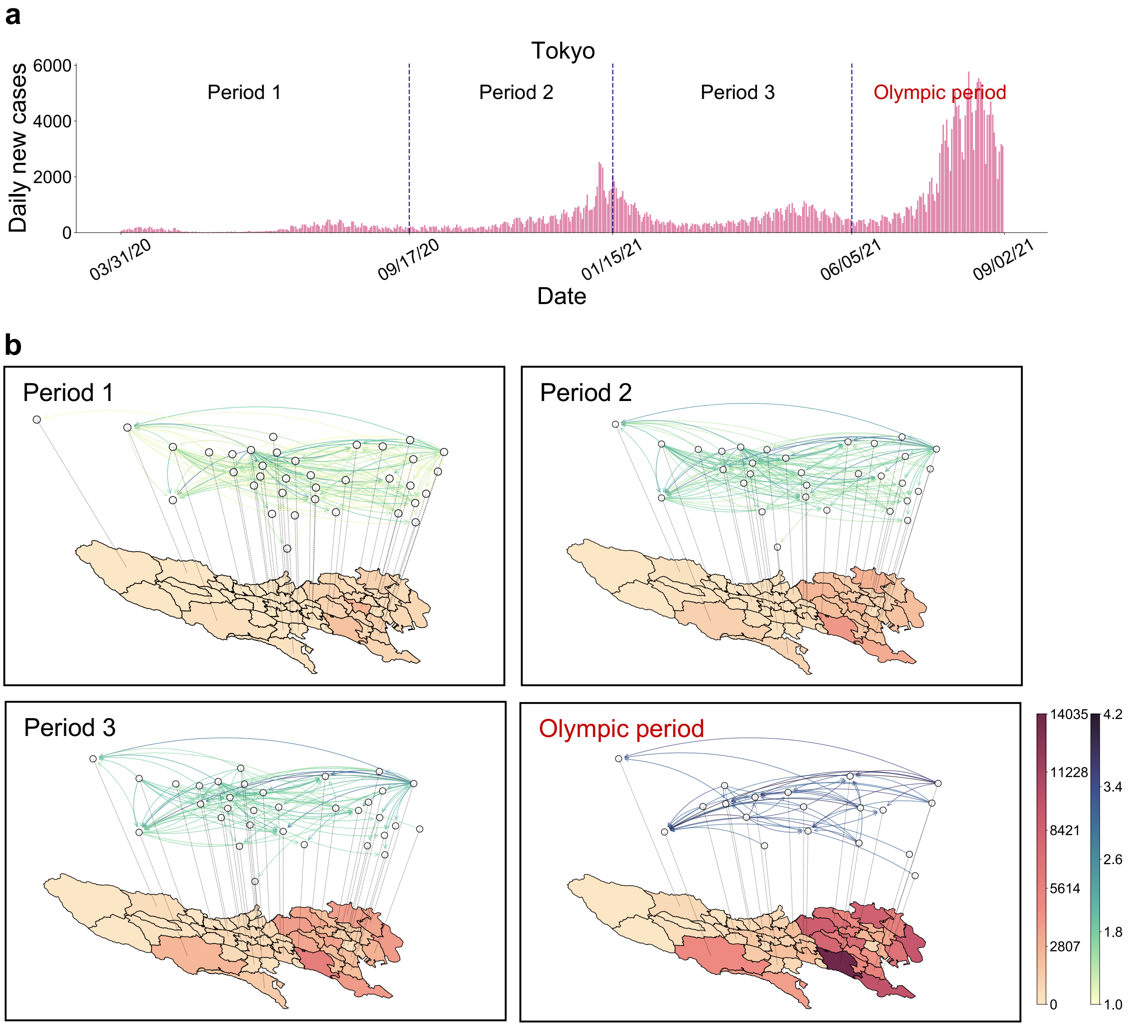

Supplementary Figure 8 ***Results of temporal clustering of the case dynamics and the inferred TransCode for each period in Tokyo. (a)*** *The sequence is partitioned into four periods, demonstrating different temporal patterns. The last segment is called the Olympic period because the Tokyo Summer Olympics were held during this period (July 23 to August 8, 2021), representing the main cause of this outbreak wave.* ***(b)*** *Visualization of the inferred TransCodes for four periods in Tokyo. In each subfigure, the map shows the number of cumulative confirmed cases in each district during the corresponding period (darker colors indicate higher case numbers); the transmission network represents the inferred TransCode. The network nodes correspond to the districts, and the directed network edges show transmission from one district to another (darker edge colors indicate higher transmission intensities).*

5.7 Hong Kong

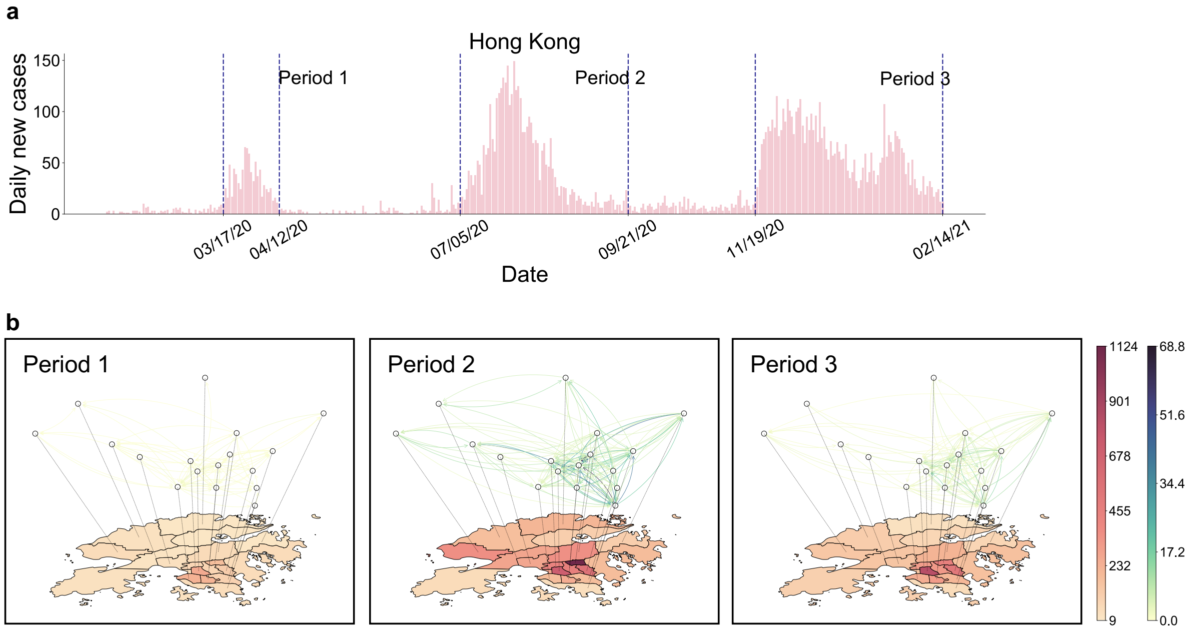

Supplementary Figure 9 ***Partition of the case dynamics and the inferred TransCode for each period in Hong Kong.*** *(a) The sequence is partitioned into three periods, demonstrating different temporal patterns. (b) Visualization of the inferred TransCodes for three periods in Hong Kong. In each subfigure, the map shows the number of cumulative confirmed cases in each district during the corresponding period (darker colors indicate higher case numbers); the transmission network represents the inferred TransCode. The network nodes correspond to the districts, and the directed network edges show transmission from one district to another (darker edge colors indicate higher transmission intensities).*

6 Supplementary references

1. David, S. B., Lu, T., Luu, T., & Pál, D., Impossibility theorems for domain adaptation. in *Proc. 13th Int. Conf. Artif. Intell.* **(129-136)** 2010.
2. Kifer, D., Ben-David, S., & Gehrke, J., Detecting Change in Data Streams, *in Proc. 30th VLDB*, **4(180-191)** 2004.
3. Ben-David, S., Blitzer, J., Crammer, K., & Pereira, F., Analysis of representations for domain adaptation, *in Proc. 19th NIPS*, **(137-144)** 2006.
4. Cai, Y., & Lim, L. H., Distance between probability distributions of different dimensions. *IEEE Trans. Inf. Theory*, 2022.
5. Goodfellow, I., Bengio, Y., & Courville, A., *Deep Learning*. (MIT press, Cambridge 2016).
